# Risk factor analysis and clinicopathological characteristics of female dogs with mammary tumours from a single-center retrospective study in Poland

**DOI:** 10.1038/s41598-024-56194-z

**Published:** 2024-03-06

**Authors:** Izabella Dolka, Michał Czopowicz, Diana Stopka, Agata Wojtkowska, Ilona Kaszak, Rafał Sapierzyński

**Affiliations:** 1 Department of Pathology and Veterinary Diagnostics, Institute of Veterinary Medicine, Warsaw University of Life Sciences (SGGW), Nowoursynowska 159C, 02-776 Warsaw, Poland; 2 Division of Veterinary Epidemiology and Economics, Institute of Veterinary Medicine, Warsaw University of Life Sciences (SGGW), Nowoursynowska 159C, 02-776 Warsaw, Poland; 3 Department of Small Animal Diseases With Clinic, Institute of Veterinary Medicine, Warsaw University of Life Sciences (SGGW), Nowoursynowska 159C, 02-776 Warsaw, Poland

**Keywords:** Dog, Mammary tumour, Histopathology, Prognosis, Ovariohysterectomy, Poland, Cancer, Risk factors

## Abstract

This is a comprehensive retrospective study to characterize female dogs with canine mammary tumors (CMTs) using a dataset retrieved from the archives of the Division of Animal Pathology, Institute of Veterinary Medicine in Warsaw, and to identify prognostic factors. Clinical and histopathological data of 1447 dogs with CMTs were included. Malignant tumours were found in 83.3% (n = 1206), benign tumours in 11.7% (n = 169), and non-neoplastic lesions in 5.0% (n = 72) of dogs. Dogs most often had grade II carcinomas (38.2%, 215/562) of a single histological subtype (88.5%, 1281/1447), mostly simple carcinoma (35.3%, 510/1447). Dogs with a median age of 10 years significantly often had larger (≥ 3 cm) and malignant CMTs, whereas intact females had smaller tumours (median size 2.0 cm). However, the threshold value for the age of the dog in the differentiation of malignant and non-neoplastic/benign masses could not be determined. Most females were hormonally active (76.4%, 372/487). Hormonally active dogs significantly more often had multiple tumours. Multiple tumours were significantly smaller (median 2.5 cm) than single ones. Among pedigree dogs, small-breed dogs were mostly recorded (43%, 428/1006). Twelve breeds had an increased risk of CMTs, regardless of tumour behaviour, compared with the theoretical distribution of pedigree dogs in Poland. Four breeds were often affected only by malignant and other four breeds only by non-neoplastic/benign CMT. Large-breed dogs were significantly younger and affected by larger CMT (median 4 cm) compared with small- and medium-breed dogs. Ninety dogs with a malignant CMT and complete records were included in the full analysis of CMT-specific survival (CMT-SS) with a median follow-up time of 20.0 months. We showed that the timing of ovariohysterectomy in relation to mastectomy was significantly associated with grade, CMT-SS, and CMT-related death. We indicated the low diagnostic accuracy of palpation of regional lymph nodes (RLN) in the prediction of their metastatic involvement. By multivariable analysis, dogs with neoplastic emboli, tumour ulceration, and simple or complex carcinoma had a significantly higher risk of local recurrence. Tumour size > 3 cm was as a strong independent predictor of lung metastases. Compared with dogs with an easily separated localized tumour, dogs with a multiple/diffuse malignant CMT pattern had a fivefold higher risk of death. The risk of death was significantly higher in the presence of neoplastic emboli (~ fivefold) and tumour ulceration (~ fourfold). Furthermore, the presence of neoplastic emboli and large tumour size were independent predictors of CMT-related death.

## Introduction

Mammary tumours are the most prevalent type of tumour in female intact dogs (*Canis familiaris*) globally^1–3^, and the second most common tumours after skin tumours in dogs^4–6^. According to a US study, the annual incidence rate for canine mammary tumours (CMTs) was 257.6 per 100,000 female dogs in California, USA (1963–1966)^4^, whereas in European studies, it was 250 per 100,000 female dogs in Italy (2005–2013)^7^, 205 per 100,000 female dogs in the population of insured dogs in the UK (1997–1998)^8^, and 1110 per 100,000 female dogs in Sweden (1995–2002)^9^. Multiple factors affect the incidence rate of CMTs, including age, breed, reproductive status, diet, and obesity^10,11^. Several studies have stated that middle-aged and older dogs (over 6 years, average range of 8–11 years), dogs of certain breeds (especially miniature and toy with possibly a genetic component), overweight dogs, those fed a diet rich in red meat, and one-year-old obese dogs, have an increased risk of developing mammary dysplasia and neoplasms^9–12^. It is well known that CMTs may be sex-steroid hormone-dependent, and according to the most cited and the earliest study, the risk of CMTs depends on the age of the dog at the time of ovariohysterectomy (OH)^13,14^. According to some authors, the well-established common claims are based on clinical observations, insufficient data, conflicting research results, and unclear statistical methodology. Attention should be drawn to the determination of the optimal age for OH (how early is too early?), also considering the adverse effects of early OH^15–17^. Moreover, a recent study suggests that OH performed in adulthood (≥ 4 years of age) may still decrease the risk of CMT development; however, a reduction in the prevalence of benign tumours has been observed in neutered dogs^18^. Even late-spayed dogs (after 2 years of age) had a fourfold lower risk of CMT-related death compared with intact dogs^17^. The prevalence of malignant CMTs varies between studies and accounts for 40% to 60%^1–3,19^ or even 70% to 90% of all CMTs^7,20–23^. Malignant CMTs were a cause of high mortality ranging from 25%^21,24^ to 42%^25^. These differences in the percentages can be due to different spaying rates in each country. If there is a high spaying rate, there will be a decreased total CMT rate but an increase in malignant tumours among the CMT^18^.

Regional lymph nodes (RLN), such as the axillary and superficial inguinal lymph nodes, are usually affected in female dogs with metastatic CMTs^26^. Histopathology remains the gold standard for diagnosis of lymph node metastases in patients with CMT, as well as in human breast cancer (HBC)^27,28^. Some studies of HBC have investigated the accuracy of clinical examination of palpable axillary lymph nodes in diagnosing metastases compared with histopathology. The overall accuracy ranged from 50 to 68%, with a diagnostic sensitivity (Se) of 30% to 64% and a diagnostic specificity (Sp) of 60% to 93%^29–31^. No such reports are available for CMTs, and, inspired by the HBC studies, we thought it would be interesting to learn more about the accuracy of the clinical status of RLN compared with the pathologic lymph node status in female dogs^31–33^.

There is no tumour registration system for dogs in Poland, and the size of the total Polish canine population remains largely unknown. According to the Kantar Public survey in 2017, 60% of households in Poland owned a dog, followed by those owning a cat (44%). Based on previous District Veterinary Inspections data, the number of dogs in Warsaw was estimated at approximately 120,000^34,35^. Despite extensive research on CMTs over the years in different regions of the world, to the best of the authors’ knowledge, the epidemiological data on dogs affected by CMTs in East-Central Europe are limited or quite old in the veterinary literature, which makes the current characteristics of patients with CMTs unclear^19,36,37^. Therefore, we decided to carry out a retrospective investigation of the CMT prevalence in female dogs over a 24-year period and to study the associations between epidemiological and clinicopathological characteristics (e.g. age, breed, reproductive status, number of tumours, location, tumour size), as well as clinical outcome – CMT-specific survival (CMT-SS) and CMT-related death.

## Material and methods

### Cover letters review

The submission letters and archived reports of female dogs diagnosed with CMT at the Division of Animal Pathology, Department of Pathology and Veterinary Diagnostics, Institute of Veterinary Medicine, Warsaw University of Life Sciences (SGGW) between January 1996 and December 2019, were retrospectively reviewed. This Division is the only University reference center for veterinary pathology covering the Masovian district (52°13′N 21°0′E) with the total area of 35,579 km^2^ and a population of 5.4 million inhabitants, and Warsaw with 1.8 million residents. The total number of female dogs with CMTs in our database in a given period was used to calculate the prevalence of CMT among samples submitted for histopathology. The case inclusion criteria consisted of female dog with CMT confirmed by histopathology. Dogs were excluded if the gender of the dog or the histological subtype of CMT was unknown. The following epidemiological data were collected: age at diagnosis (< 8 years / ≥ 8 years); pedigree of the dog (mixed/pedigree); height of a pedigree dog based on withers height according to the Féderation Cynologique International (FCI) nomenclature (small up to 35 cm, medium 35–50 cm, large over 50 cm)^3,38^; FCI groups; spay status at mastectomy (spayed at any time before mastectomy/intact = never spayed or spayed during mastectomy); timing of OH (OH at the time of mastectomy/OH < 1 year before mastectomy but not concurrent with mastectomy/OH ≥ 1 year before mastectomy); hormonal status at mastectomy (hormonally active = never spayed, OH during mastectomy, OH < 1 year before mastectomy)/hormonally inactive = OH ≥ 1 year before mastectomy); regular oestrous; previous hormonal contraception; pseudopregnancy; pyometra/mucometra; number of tumours in a dog (single/multiple = two or more tumours); defined separate, localized/multiple tumours, diffuse pattern with unclear tumour boundaries; side (right/left/both); location of affected glands; tumour size (clinical entire tumour size based on gross measurements or after making a cross-section of the tumour, the largest diameter of tumour in centimeter, < 3 cm / ≥ 3 cm); biological behaviour of tumour (non-neoplastic lesion/neoplastic tumours: benign, malignant); histological subtype (simple carcinoma/complex carcinoma/other malignant subtypes); histological grade of malignancy (I, II, III); presence of neoplastic emboli (presence of neoplastic cell clusters within lymphatic vessels at the periphery of the neoplasm, the feature of lymphovascular invasion); ulceration of the skin above the tumour (based on clinical examination and/or histopathology); tumour necrosis (based on histopathology); clinical TNM staging^26^; RLN metastases determined by histopathology at the time of diagnosis; enlarged RLN (based on clinical examination); lung metastases investigated by thoracic radiography at the time of diagnosis.

After mastectomy, dogs were followed up for at least 24 months. They were censored if they died from causes unrelated to CMT, or were still alive at the end of the observation period. In this study, each dog was counted only once even if it appeared several times in our database over the years, and only its CMTs were included.

In the case of dogs with more than one malignant CMT, the one with the worst tumour behaviour (based on histopathology) was selected for statistical analysis (e.g. a dog with a non-neoplastic lesion or a benign tumour and a malignant tumour was classified as a dog with a malignant tumour. A dog with a non-neoplastic lesion and a benign tumour was classified as a dog with a benign tumour).

### Follow-up

We used survival data collected over many years, not only over several years preceding the study. The 2-year follow-up data were obtained through a telephone interview (survey) with dog owner and/or contact with the referring veterinarians, and/or were retrieved from the medical records. The follow-up data of cases from 1996 to 2005 were unavailable due to the lack of contact options (e.g. no telephone number, no e-mail address, unsuccessful attempt to deliver the survey to the address given), and/or unavailable or incomplete medical records. The following data were recorded: local recurrence, lung metastases in thoracic radiography, CMT-specific survival (CMT-SS) defined as the time from the date of mastectomy to the date of CMT-related death, and CMT-related death refers to death attributable to malignant CMT.

### Histopathology

Tumour samples were fixed in 10% neutral buffered formalin immediately after collection, then routinely processed and stained with haematoxylin and eosin (H-E). Additionally, some cases were stained with Masson, Van Gieson, Periodic acid-Schiff (PAS), Mucicarmin, and Sudan. CMTs were classified into subtypes following the 2011 classification^39^ and the Peña grading system^40^. If histological grade was established based on the grading numeric system known as the Elston and Ellis method adapted to CMTs^41^, this grade was retained. Based on the availability of archival paraffin blocks, cases were reevaluated and, if required, immunohistochemistry (IHC) for Pan-cytokeratin, vimentin, αSMA, desmin, and p63 was performed as we described elsewhere^42,43^. Risk factors of local recurrence, regional lymph node and lung metastases were calculated for malignant CMT.

### Statistical methods

Numerical variables were presented as the median, interquartile range (IQR), and range, and they were compared between groups with the Mann–Whitney U test (2 groups) or with the Kruskal–Wallis test (> 2 groups). Categorical variables were presented as a count and percentage in a group and compared between groups with the maximum likelihood G test or Fisher’s exact test. Trends in proportions were examined using the χ2 test for trends. The 95% confidence intervals (CI) for proportions were calculated using the Wilson score method. Diagnostic accuracy was investigated using the area under ROC curve (AUROC) analysis, and diagnostic sensitivity (Se) and specificity (Sp) were reported. Risk factors of local recurrence, lung metastases, and death for which the *p* value was below 0.1 in univariable analysis, were introduced into multivariable analysis based on the multiple logistic regression model (backward elimination) or Cox proportional-hazard model (in terms of survival analysis). Size of effect was expressed as adjusted odds ratio (OR_adj_) or adjusted hazard ratio (HR_adj_) with CI 95%. All statistical tests were two-tailed. The significance level (α) was set at 0.05. Statistical analysis was performed in TIBCO Statistica 13.3 (TIBCO Software Inc., Palo Alto, CA, USA).

### Ethical approval

The samples were submitted by veterinary clinical practitioners between January 1996 and December 2019 after routine therapeutic mastectomy. Therefore, approval of II Local Ethics Committee for Animal Experiments in Warsaw University of Life Sciences was not required for this study according to the Act of 15 January 2015 on protection animals used for scientific or educational purposes (Journal of Laws of 2015, item 266) and subsequent amendments (Journal of Laws of 2021, item 2338), implementing the Directive 2010/63/EU. The use of data from retrospective records for research purposes was allowed by the Institute of Veterinary Medicine, Warsaw University of Life Sciences. The owners granted a written permission for taking tissue samples for histopathology. All methods were performed in accordance with relevant guidelines/regulations in the Institute of Veterinary Medicine, Warsaw University of Life Sciences. The study was carried out in compliance with the ARRIVE guidelines.

### Institutional animal care and use committee (IACUC) or other approval declaration

Authors declare no IACUC or other approval was needed.

### Human ethics approval declaration

Authors declare human ethics approval was not needed for this study

## Results

### Characteristics of the population

#### Age and breed

CMT was diagnosed in 1447 female dogs aged from 1 to 17 years with a median (IQR) of 10 (8–12) years. Dogs with malignant CMT were significantly older (median 10 years, IQR 8–12 years, range 1–17 years) than dogs with benign tumours (median 9 years, IQR 7–10 years, range 2–16 years, *p* < 0.001) or non-neoplastic lesions (median 8 years, IQR 7–10 years, range 2.5–14.5 years, *p* = 0.002). Although the age significantly differed between these 2 groups (*p* < 0.001), its discriminatory potential was low (AUROC = 60.7%; 95% CI 56.7%–64.7%) (Supplementary Table 1). Young dogs (at age ≤ 5 years) with CMTs accounted for 5.8% (79/1371). Young dogs ≤ 5 years (79/1371) had significantly more often non-neoplastic or benign tumours (*p* = 0.001) (Supplementary Table 2). The main characteristics of dogs and their CMTs are given in Table 1.

**Table 1:** Epidemiological and clinicopathological characteristics of all 1447 dogs with mammary tumours.

Characteristics [Number of dogs (% of all 1447 dogs) with available data]	Number (%) of dogs
Age [1371 (94.8)]	Median 10 years, IQR 8–12 years (range 1–17 years)
Breed [1381 (95.4)]	
Mixed-breed	375 (27.2)
Pedigree	1006 (72.8)
Small^a^	428 (42.5)
Medium	172 (17.1)
Large	406 (40.4)
Spay status at time of mastectomy [487]	
Intact = never spayed or spayed during mastectomy	343 (70.4)
OH with mastectomy	241 (70.3)
Only mastectomy	102 (29.7)
Spayed	144 (29.6)
The timing of OH in female dogs [110]	
OH < 1 year before mastectomy	29 (26.4)
OH ≥ 1 year before mastectomy	81 (73.6)
Hormonal status at mastectomy [453 (31.3%)]	
Active = never spayed or spayed during mastectomy, OH < 1 year before mastectomy	372 (82.1)
Inactive = OH ≥ 1 year before mastectomy	81 (17.9)
Regular oestrus [33 (2.3)]	
Yes	32
No	1
Hormonal contraception [101 (7.0)]	
Yes	11
No	90
Pseudopregnancy [53 (3.7)]	
Yes	36
No	17
Pyometra/mucometra [44 (3.0)]	
Yes	44
No or unknown	1403

IQR: interquartile range; OH: ovariohysterectomy.

^a^Pedigree dog’s height based on withers height according to the Féderation Cynologique International (FCI) nomenclature.

Pedigree dogs (72.8%, 1006/1381) represented by 100 breeds, including those not classified by the FCI, outnumbered mixed-breed dogs (27.2%, n = 375) (Supplementary Table 3). Small-breed dogs (43%, n = 428) were most common, followed by large-breed dogs (40%, n = 406), and medium-breed dogs (17%, n = 172). Based on the theoretical distribution of female pedigree dogs in Poland according to available registers of the Polish Kennel Club from years 2009–2019^44^, 12 breeds were significantly overrepresented among dogs with CMT of all tumour behaviour: Standard Dachshund, German Shepherd Dog, Yorkshire Terrier, Boxer, English Cocker Spaniel, Miniature Poodle, Doberman, Standard Schnauzer, Miniature Pinscher, Giant Schnauzer, Fox Terrier, and Medium Poodle and fifteen breeds were significantly under-represented: Labrador Retriever, French Bulldog, West Highland White Terrier, Polish Hunting Dog, Siberian Husky, Chihuahua, Bull Terrier, Central Asia Shepherd Dog, English Bulldog, Cavalier King Charles Spaniel, Maltese, Basset Hound, Pug, Border Collie, and Italian Cane Corso (Supplementary Table 4). Moreover, breeds classified into group 4 (Dachshunds) according to the FCI^38^ were significantly overrepresented, whereas breeds classified into group 5 (Spitz and primitive types) and group 9 (Companion and Toy Dogs) were significantly underrepresented among dogs with CMT (Table 2). German Shepherd Dog, Standard Schnauzer, Giant Schnauzer, and Medium Poodle turned out to be overrepresented only among dogs with malignant CMTs, whereas Labrador Retriever, French Bulldog, Jack Russel Terrier, Italian Cane Corso, Polish Hunting Dog, Chihuahua, English Bulldog, Cavalier King Charles Spaniel, and Maltese were significantly underrepresented among dogs with malignant CMT. Fox Terrier, Beagle, Pekingese, Black Russian Terrier were overrepresented only among dogs with non-neoplastic/benign CMT (Table 3). Large-breed dogs with CMT (regardless of tumour behaviour) as well as with malignant CMT were significantly younger (median age 9.0 years, *p* < 0.001) than small- and medium-breed dogs (median age 10 years). Large breeds presented with significantly larger CMT in general (median size 4.0 cm, *p* < 0.001) compared with small and medium breeds (median size 2.0 cm). The difference was similar for malignant CMT (median size 4.0 cm, 2.0 cm, 2.3 cm for large, small, and medium breeds, respectively, *p* < 0.001).

**Table 2:** Relationship between FCI group and the occurrence of CMT in pedigree dogs.

FCI group	CMT [n (%)]		OR (95% CI)	*p* value
	Female pedigree dogs affected with CMT (n = 1002)^c^	Polish theoretical distribution of 1000 female pedigree dogs		
1	169 (16.9)	166 (16.6)	1.02 (0.81–1.29)	0.873
Sheepdogs and Cattledogs (except Swiss Cattledogs)				
2	220 (22)	206 (20.6)	1.08 (0.88–1.34)	0.459
Pinscher and Schnauzer—Molossoid and Swiss Mountain and Cattledogs				
3	169 (16.9)	164 (16.4)	1.03 (0.82–1.31)	0.779
Terriers				
4	194 (19.4)	28 (2.8)	8.33 (5.55–12.5)	< 0.001*
Dachshunds^a^				
5	17 (1.7)	65 (6.5)	0.25 (0.14–0.43)	< 0.001*
Spitz and primitive types^b^				
6	40 (4.0)	56 (5.6)	0.70 (0.46–1.06)	0.092
Scent hounds and related breeds				
7	29 (2.9)	30 (3.0)	0.96 (0.57–1.62)	0.889
Pointing Dogs				
8	76 (7.6)	94 (9.4)	0.79 (0.58–1.08)	0.145
Retrievers-Flushing Dogs- Water Dogs				
9	76 (7.6)	168 (16.8)	0.41 (0.31–0.54)	< 0.001*
Companion and Toy Dogs^b^				
10	12 (1.2)	22 (2.2)	0.54 (0.27–1.09)	0.080
Sighthounds				

^a^FCI groups significantly overrepresented (suspected predisposition to CMT),

^b^FCI groups significantly under-represented,

^c^Four breeds were not classified by FCI,

CMT: canine mammary tumour; FCI Féderation Cynologique International; OR: crude odds ratio; CI confidence interval,

*Significant at α = 0.05.

**Table 3:** Distribution and calculation of ORs of malignant CMT, non-neoplastic lesions and benign CMT based on the theoretical distribution of Polish pedigree dogs based on registers of the Polish Kennel Club from years 2009–2019.

Breed	CMT [n (%)]			Malignant	*p* value	Benign	*p* value
	Polish theoretical distribution of 1000 female pedigree dogs	Female pedigree dogs affected with malignant CMT (n = 833)	Female pedigree dogs affected with Non-neoplastic lesions or benign CMT (n = 173)	OR (95% CI)		OR (95% CI)	
Standard Dachshund^a^	28 (2.8)	166 (19.9)	28 (16.2)	8.64 (5.72–13.1)	< 0.001*	6.70 (3.86–11.6)	< 0.001*
German Shepherd Dog^a^	86 (8.6)	126 (15.1)	16 (9.2)	1.89 (1.42–2.53)	< 0.001*	1.08 (0.62–1.90)	0.781
Yorkshire Terrier^a^	50 (5.0)	73 (8.8)	22 (12.7)	1.83 (1.26–2.65)	0.001*	2.77 (1.63–4.70)	< 0.001*
Boxer^a^	8 (0.8)	40 (4.8)	10 (5.8)	6.25 (2.91–13.4)	< 0.001*	7.61 (2.96–19.6)	< 0.001*
English Cocker Spaniel^a^	7 (0.7)	36 (4.3)	6 (3.5)	6.41 (2.84–14.5)	< 0.001*	5.10 (1.69–15.4)	0.007*
Miniature Poodle^a^	4 (0.4)	26 (3.1)	8 (4.6)	8.02 (2.79–23.1)	< 0.001*	12.1 (3.59–40.6)	< 0.001*
Doberman^a^	6 (0.6)	24 (2.9)	8 (4.6)	4.91 (2.00–12.1)	< 0.001*	8.03 (2.75–23.5)	< 0.001*
Standard Schnauzer^a^	3 (0.3)	26 (3.1)	2 (1.2)	10.7 (3.23–35.5)	< 0.001*	3.89 (0.64–23.4)	0.169
Miniature Schnauzer	19 (1.9)	23 (2.8)	3 (1.7)	1.47 (0.79–2.71)	0.221	0.91 (0.27–3.11)	0.881
American Staffordshire Terrier	13 (1.3)	17 (2)	5 (2.9)	1.58 (0.76–3.28)	0.214	2.26 (0.80–6.42)	0.152
Golden Retriever	22 (2.2)	17 (2)	3 (1.7)	0.93 (0.49–1.76)	0.814	0.78 (0.23–2.65)	0.687
Rottweiler	10 (1.0)	17 (2)	1 (0.6)	2.06 (0.94–4.53)	0.066	0.58 (0.07–4.53)	0.572
Miniature Pinscher^a^	4 (0.4)	13 (1.6)	4 (2.3)	3.95 (1.28–12.2)	0.009*	5.89 (1.46–23.8)	0.018*
Giant Schnauzer^a^	5 (0.5)	15 (1.8)	–	3.65 (1.32–10.1)	0.007*	–	–
Fox Terrier^a^	4 (0.4)	10 (1.2)	4 (2.3)	3.03 (0.95–9.68)	0.091	5.89 (1.46–23.8)	0.018*
Weimaraner	6 (0.6)	10 (1.2)	2 (1.2)	2.01 (0.73–5.56)	0.169	1.94 (0.39–9.68)	0.447
Bavarian Mountain Scent Hound	8 (0.8)	10 (1.2)	2 (1.2)	1.51 (0.59–3.84)	0.388	1.45 (0.31–6.89)	0.652
Beagle	12 (1.2)	5 (0.6)	6 (3.5)	0.50 (0.17–1.42)	0.174	2.96 (1.10–7.99)	0.047*
Labrador Retriever^b^	40 (4.0)	8 (1)	3 (1.7)	0.23 (0.11–0.50)	< 0.001*	0.42 (0.13–1.38)	0.109
French Bulldog^b^	29 (2.9)	9 (1.1)	–	0.37 (0.17–0.78)	0.005*	–	–
Medium Poodle^a^	1 (0.1)	7 (0.8)	1 (0.6)	8.47 (1.04–69.0)	0.012*	5.81 (0.36–93.3)	0.240
Irish Red Setter	5 (0.5)	4 (0.5)	3 (1.7)	0.96 (0.26–3.59)	0.952	3.51 (0.83–14.8)	0.113
Collie (Rough)	6 (0.6)	7 (0.8)	–	1.40 (0.47–4.19)	0.543	–	
Pekingese	1 (0.1)	4 (0.5)	3 (1.7)	4.82 (0.54–43.2)	0.112	17.6 (1.82–170)	0.007*
Great Dane	13 (1.3)	4 (0.5)	2 (1.2)	0.37 (0.12–1.13)	0.060	0.89 (0.20–3.97)	0.875
Shih Tzu	8 (0.8)	4 (0.5)	2 (1.2)	0.60 (0.18–1.99)	0.392	1.45 (0.31–6.89)	0.652
Black Russian Terrier	6 (0.6)	2 (0.2)	4 (2.3)	0.40 (0.08–1.98)	0.231	3.92 (1.09–14.0)	0.051*
West Highland White Terrier^b^	16 (1.6)	5 (0.6)	1 (0.6)	0.37 (0.14–1.02)	0.075	0.36 (0.05–2.71)	0.246
Jack Russel Terrier	15 (1.5)	2 (0.2)	3 (1.7)	0.16 (0.04–0.69)	0.003*	1.16 (0.33–4.05)	0.820
Italian Sighthound	4 (0.4)	5 (0.6)	–	1.50 (0.40–5.62)	0.543	–	–
Gordon Setter	3 (0.3)	4 (0.5)	1 (0.6)	1.60 (0.36–7.19)	0.534	1.93 (0.20–18.7)	0.592
Alaskan Malamute	6 (0.6)	4 (0.5)	1 (0.6)	0.80 (0.22–2.84)	0.728	0.96 (0.12–8.05)	0.972
Dalmatian	1 (0.1)	4 (0.5)	1 (0.6)	4.82 (0.54–43.2)	0.112	5.81 (0.36–93.3)	0.240
Staffordshire Bull Terrier	11 (1.1)	4 (0.5)	1 (0.6)	0.43 (0.14–1.37)	0.133	0.52 (0.07–4.07)	0.498
Polish Hunting Dog^b^	13 (1.3)	3 (0.4)	1 (0.6)	0.27 (0.08–0.97)	0.024*	0.44 (0.06–3.40)	0.377
Siberian Husky^b^	13 (1.3)	4 (0.5)	–	0.37 (0.12–1.13)	0.060	–	–
Polish Lowland Sheepdog	6 (0.6)	3 (0.4)	1 (0.6)	0.60 (0.15–2.40)	0.459	0.96 (0.12–8.05)	0.972
Pembroke Welsh Corgi	3 (0.3)	3 (0.4)	1 (0.6)	1.20 (0.24–5.97)	0.823	1.93 (0.20–18.7)	0.592
Akita–Akita Inu	8 (0.8)	3 (0.4)	1 (0.6)	0.45 (0.12–1.69)	0.214	0.72 (0.09–5.80)	0.748
Chihuahua^b^	37 (3.7)	3 (0.4)	1 (0.6)	0.09 (0.03–0.31)	< 0.001*	0.15 (0.02–1.11)	0.056
American Pit Bull Terrier^c^		3 (0.4)	–	–	–	–	–
Bull Terrier^b^	12 (1.2)	3 (0.4)	–	0.30 (0.08–1.06)	0.084	–	–
Bedlington Terrier	1 (0.1)	2 (0.2)	1 (0.6)	2.40 (0.22–26.6)	0.459	5.81 (0.36–93.3)	0.240
Briard	3 (0.3)	3 (0.4)	–	1.20 (0.24–5.97)	0.823	–	–
Airedale Terrier	4 (0.4)	2 (0.2)	1 (0.6)	0.60 (0.11–3.28)	0.546	1.45 (0.16–13.0)	0.751
Welsh Terrier	4 (0.4)	3 (0.4)	–	0.90 (0.20–4.03)	0.890	–	–
German Wirehaired Pointer	2 (0.2)	2 (0.2)	1 (0.6)	1.20 (0.17–8.54)	0.855	2.90 (0.26–32.2)	0.420
Whippet	7 (0.7)	3 (0.4)	–	0.51 (0.13–1.99)	0.316	–	–
Rhodesian Ridgeback	4 (0.4)	3 (0.4)	–	0.90 (0.20–4.03)	0.890	–	–
Afghan Hound	1 (0.1)	2 (0.2)	–	2.40 (0.22–26.6)	0.459	–	–
Old English Sheepdog (Bobtail)	1 (0.1)	2 (0.2)	–	2.40 (0.22–26.6)	0.459	–	–
Bullmastiff	2 (0.2)	1 (0.1)	1 (0.6)	0.60 (0.05–6.63)	0.669	2.90 (0.26–32.17)	0.420
Bouvier des Flandres	1 (0.1)	2 (0.2)	–	2.40 (0.22–26.6)	0.459	–	–
Scottish Terrier	4 (0.4)	2 (0.2)	–	0.60 (0.11–3.28)	0.546	–	–
Irish Red Terrier	1 (0.1)	1 (0.1)	1 (0.6)	1.20 (0.07–19.2)	0.897	5.81 (0.36–93.3)	0.240
Caucasian Shepherd Dog	6 (0.6)	2 (0.2)	–	0.40 (0.08–1.98)	0.231	–	–
Bloodhound	1 (0.1)	1 (0.1)	1 (0.6)	1.20 (0.07–19.23)	0.897	5.81 (0.36–93.3)	0.240
American Akita	4 (0.4)	1 (0.1)	1 (0.6)	0.30 (0.03–2.68)	0.233	1.45 (0.16–13.0)	0.751
Central Asia Shepherd Dog^b^	10 (1.0)	2 (0.2)	–	0.24 (0.05–1.09)	0.086	–	–
English Bulldog^b^	18 (1.8)	1 (0.1)	1 (0.6)	0.07 (0.01–0.49)	< 0.001*	0.32 (0.04–2.39)	0.185
St.Bernard	7 (0.7)	2 (0.2)	–	0.34 (0.07–1.65)	0.146	–	–
Polish Hound	6 (0.6)	2 (0.2)	–	0.40 (0.08–1.98)	0.231	–	–
Hovawart	9 (0.9)	2 (0.2)	–	0.27 (0.06–1.23)	0.056	–	–
Tatra Shepherd Dog	1 (0.1)	2 (0.2)	–	2.40 (0.22–26.6)	0.459	–	–
Cavalier King Charles Spaniel^b^	18 (1.8)	2 (0.2)	–	0.13 (0.03–0.57)	0.001*	–	–
Maltese^b^	12 (1.2)	2 (0.2)	–	0.20 (0.04–0.89)	0.012*	–	–
Dog de Bordeaux	4 (0.4)	1 (0.1)	–	0.30 (0.03–2.68)	0.233	–	–
Leonberger	5 (0.5)	1 (0.1)	–	0.24 (0.03–2.05)	0.135	–	–
Tosa–Tosa Inu	3 (0.3)	1 (0.1)	–	0.40 (0.04–3.85)	0.398	–	-
Cairn Terrier	3 (0.3)	1 (0.1)	–	0.40 (0.04–3.85)	0.398	–	–
Irish Soft Coated Wheaten Terrier	1 (0.1)	1 (0.1)	–	1.20 (0.07–19.2)	0.897	–	–
German Giant Spitz^c^		1 (0.1)	–			–	–
Tibetan Terrier	1 (0.1)	1 (0.1)	–	1.20 (0.07–19.2)	0.897	–	–
Russian-European Laika	2 (0.2)	1 (0.1)	–	0.60 (0.05–6.63)	0.669	–	–
Flat Coated Retriever	5 (0.5)	1 (0.1)	–	0.24 (0.03–2.05)	0.135	–	–
Basset Hound^b^	8 (0.8)	(0)	1 (0.6)	–	–	0.72 (0.09–5.80)	0.748
American Cocker Spaniel	1 (0.1)	(0)	1 (0.6)	–	–	5.81 (0.36–93.3)	0.240
German Pinscher	7 (0.7)	1 (0.1)	–	0.17 (0.02–1.39)	0.127	–	–
German Hunting Terrier, Deutcher Jagdterier	7 (0.7)	1 (0.1)	–	0.17 (0.02–1.39)	0.127	–	–
Bichon Frisé	3 (0.3)	1 (0.1)	–	0.40 (0.04–3.85)	0.398	–	–
Newfoundland	7 (0.7)	1 (0.1)	–	0.17 (0.02–1.39)	0.127	–	–
Norwich Terrier	1 (0.1)	1 (0.1)	–	1.20 (0.07–19.2)	0.897	–	–
Lhasa Apso	4 (0.4)	1 (0.1)	–	0.30 (0.03–2.68)	0.233	–	–
Great Swiss Mountain Dog	4 (0.4)	1 (0.1)	–	0.30 (0.03–2.68)	0.233	–	–
Shar Pei	4 (0.4)	1 (0.1)	–	0.30 (0.03–2.68)	0.233	–	–
Continental Toy Spaniel (Papillon)	6 (0.6)	1 (0.1)	–	0.20 (0.02–1.66)	0.077	–	–
Australian Terrier	1 (0.1)	1 (0.1)	–	1.20 (0.07–19.2)	0.897	–	–
Bohemian Wire-Haired Pointing Griffon (Cesky Fousek)	1 (0.1)	1 (0.1)	–	1.20 (0.07–19.2)	0.897	–	–
English Springer Spaniel	3 (0.3)	1 (0.1)	–	0.40 (0.04–3.85)	0.398	–	–
Russian Spaniel^c^		1 (0.1)	–			–	–
Beauceron	1 (0.1)	1 (0.1)	–	1.20 (0.07–19.2)	0.897	–	–
Pug^b^	12 (1.2)	(0)	1 (0.6)			0.48 (0.06–3.71)	0.434
Presa Canario	1 (0.1)	1 (0.1)	–	1.20 (0.07–19.2)	0.897	–	–
English Setter (Laverack)	2 (0.2)	1 (0.1)	–	0.60 (0.05–6.63)	0.669	–	–
Border Collie^b^	13 (1.3)	(0)	1 (0.6)			0.44 (0.06–3.40)	0.377
Tibetan Mastiff	7 (0.7)	1 (0.1)	–	0.17 (0.02–1.39)	0.127	–	–
Czechoslovakian Wolfdog	3 (0.3)	1 (0.1)	–	0.40 (0.04–3.85)	0.398	–	–
Irish Wolfhound	2 (0.2)	1 (0.1)	–	0.60 (0.05–6.63)	0.669	–	–
Italian Cane Corso^b^	10 (1.0)	1 (0.1)	–	0.12 (0.02–0.93)	0.008*	–	–
Borzoi – Russian Hunting Sighthound	3 (0.3)	1 (0.1)	–	0.40 (0.04–3.85)	0.398	–	–

Breeds (n = 11) significantly overrepresented among dogs with malignant CMT: Standard Dachshund, German Shepherd Dog, Yorkshire Terrier, Boxer, English Cocker Spaniel, Miniature Poodle, Doberman, Standard Schnauzer, Miniature Pinscher, Giant Schnauzer, Medium Poodle.

Breeds (n = 11) were significantly overrepresented among dogs with non-neoplastic/benign CMT: Standard Dachshund, Yorkshire Terrier, Boxer, English Cocker Spaniel, Miniature Poodle, Doberman, Miniature Pinscher, Fox Terrier, Beagle, Pekingese, Black Russian Terrier (the latter one on the borderline of statistical significance).

Breeds (n = 9) significantly underrepresented among dogs with malignant CMT: Labrador Retriever, French Bulldog, Jack Russel Terrier, Italian Cane Corso, Polish Hunting Dog, Chihuahua, English Bulldog, Cavalier King Charles Spaniel, Maltese.

^a^Breeds significantly overrepresented (suspected predisposition to CMT); details in Supplementary Table 4.

^b^Breeds significantly under-represented (suspected predisposition to CMT); details in Supplementary Table 4.

^c^Not recognized as a breed by FCI,

CMT: canine mammary tumour; OR: crude odds ratios; CI confidence interval,

*Significant at α = 0.05.

#### Spay status

The hormonal/spay status was known for only 487 out of 1447 dogs (Table 1). Most dogs were hormonally active (76.4%, 372/487) and intact at day of diagnosis (70.4%, 343/487), most of them (70.3%, 241/343) underwent OH at the same time with mastectomy. Only 29.6% (144/487) of dogs were spayed. Among dogs with known timing of OH, 73.6% (81/110) of dogs were spayed more than 1 year before mastectomy, and 26.4% (29/110) of them were spayed less than 1 year beforehand. At day of mastectomy, only 17.9% (81/453) of bitches were hormonally inactive, whereas 82.1% (372/453) of dogs were hormonally active, i.e. never spayed, spayed during mastectomy, or spayed < 1 year before mastectomy (see Table 1). Dogs with CMTs had regular oestrus (96.9%, 32/33), did not use hormonal contraception (89.1%, 90/101), had experienced pseudopregnancy (67.9%, 36/53), pyometra/mucometra (n = 44).

#### Timing of OH vs. incidence of malignant tumours and tumour grade

The timing of OH in female dogs (< 1 year before mastectomy / ≥ 1 year before mastectomy) and hormonal status (hormonally active/hormonally inactive) had no significant impact on the prevalence of malignant CMTs (*p* = 0.154 and *p* = 0.215, respectively). Dogs spayed ≥ 1 year before mastectomy had grade I CMTs significantly less often and grade III CMTs significantly more often than those which underwent OH less than 1 year before mastectomy or during mastectomy (*p* = 0.002). The longer was the time elapsed from OH, the lower was the proportion of grade I CMT (*p* = 0.006), while the proportion of grade III CMT was significantly higher (*p* = 0.001). The proportion of grade II carcinomas remained stable (chi-square for trends: *p* = 0.741). The characteristics of mammary tumours in all 1447 dogs are presented in Table 4.

**Table 4:** Canine mammary tumours characteristics in all 1447 dogs.

Tumour characteristics [Number of dogs (% of all 1447 dogs) with available data]	Number (%) of dogs
Biological behavior of tumour [1447 (100)]	
Non-neoplastic lesion	72 (5.0)
Benign	169 (11.7)
Malignant	1206 (83.3)
Only malignant	1154 (95.7)
Malignant & other behaviour of tumour^a^	52 (4.3)
No. of different histological subtypes^b^	
1	1281 (88.5)
2	134 (9.3)
3	25 (1.7)
4	7 (0.5)
Number of tumours [1447 (100)]	
1	1062 (73.4)
2 and more	385 (26.6)
2	180 (12.4)
3	46 (3.2)
4	10 (0.69)
5	2 (0.14)
8	1 (0.07)
Multiple/diffuse pattern with unclear tumour boundaries	146 (10.1)
Defined separate/localized	1301 (89.9)
Side [816 (56.4)]	
Right	334 (40.9)
Left	422 (51.7)
Both	60 (7.4)
Location [872 (60.3)]	
I-II	105 (12.0)
III	81 (9.3)
IV-V	496 (56.9)
II-III	16 (1.8)
I-III	20 (2.3)
III-IV	6 (7.6)
I,II,III,IV,V	88 (10.1)
TNM [161 (11.1)]	
1	64 (39.8)
2	33 (20.5)
3	15 (9.3)
4	47 (29.2)
5	2 (1.2)
Grade of carcinoma [562 (38.8)]	
I	174 (31.0)
II	215 (38.2)
III	173 (30.8)
Neoplastic emboli [1447 (100)]	
Yes	74 (5.1)
No	1373 (94.9)
Tumour ulceration [1447 (100)]	
Yes	50 (3.5)
No	1397 (96.5)
Tumour necrosis [1447 (100)]	
Yes	392 (27.1)
No	1055 (72.9)

^a^Dogs having at least 2 tumours – one malignant and the other benign or non-malignant,

^b^166 dogs with ≥ 2 histological subtypes of CMT,

TNM: the tumour, lymph node, metastasis staging system.

#### OH and age

Age at OH was available for 335 dogs. Most were spayed with a median age of 10 years (IQR 8–12 years, range 2–16 years). Median age of OH was 10 years (IQR 7–11 years, range 0.5–16 years) for dogs spayed during mastectomy, 9.4 years (IQR 8.6–11 years, range 7–14.1 years) for dogs spayed < 1 year before mastectomy, and 7 years (IQR 3–9.7 years, range 0.5–15 years) for dogs spayed ≥ 1 year before mastectomy. Dogs spayed ≥ 1 year before mastectomy were significantly older (median 11 years, IQR 9–12 years, range 4–16 years) than dogs never spayed (median 9 years, IQR 7–11 years, range 2–15 years, *p* = 0.038) and dogs spayed during mastectomy (median 10 years, IQR 8–11 years, range 2–16 years, *p* = 0.009). No significant difference was found between dogs spayed ≥ 1 year and < 1 year before mastectomy (median 11 years, IQR 9–12 years, range 4–16 years, *p* = 0.744). Age at OH did not differ significantly between spayed dogs with malignant, benign, and non-neoplastic lesions (*p* = 0.566). (Supplementary Table 5).

#### Tumour behaviour, histological subtype, grade, and other characteristics

Most dogs had malignant tumours (83.3%, n = 1206), followed by benign tumours (11.7%, n = 169), and non-neoplastic lesions (5%, n = 72). The summary is given in Table 4. Among all dogs with malignant CMTs, 95.7% (n = 1154) of dogs had only malignant tumours, whereas 4.3% (n = 52) of dogs had the combination of tumours, i.e. one malignant and the other benign or non-neoplastic lesions (hyperplasia/dysplasia). Among all dogs, one histological subtype was recognized most frequently (88.5%, 1281/1447). Coexistence of two or more different subtypes was reported in 11.5% of dogs (166/1447), with two different subtypes occurring most commonly (9.3%, 134/1447) (Table 4). Most dogs had simple carcinomas (35.3%; 510/1447), followed by complex carcinomas (13.3%; 193/1447) and other malignant tumours (e.g. solid carcinoma, carcinoma arising in a complex/mixed special types or mesenchymal subtypes) in 51.4% of dogs (n = 744). In 1206 dogs with malignant tumours, the most common tumour subtype was simple carcinoma (n = 570; 47.3%), followed by carcinoma arising in BMT (n = 238; 19.7%), complex carcinoma (n = 233; 19.3%), and solid carcinoma (n = 52; 4.3%). In 169 dogs with benign tumours, benign mixed tumor (BMT) (n = 51; 30.2%) occurred most frequently, then simple adenoma (n = 41; 24.3%) and complex adenoma (n = 37; 21.9%). Among sarcomas, fibrosarcoma predominated (n = 23; 1.9%). With regard to the grading of carcinomas, 38.2% of dogs had grade II (215/562), followed by grade I (31.0%, 174/562) and grade III (30.8%, 173/562). Most dogs had clinical stage TNM I (39.8%, 64/161), then stage IV (29.2%, n = 47), stage II (20.5%, n = 33), stage III (9.3%, n = 15), and stage V (1.2%, n = 2). Due to the low number of complete TNM cases, this factor could not be included in multivariable analysis. The presence of tumour necrosis, neoplastic emboli, and tumour ulceration were recorded in 27.1% (392/1447), 5.1% (74/1447), and 3.5% (50/1447) of dogs, respectively.

#### Tumour size

Dogs with tumours ≥ 3 cm (n = 327) were significantly older (median age 10 years, IQR 8–12 years, range 1–17 years) than dogs with tumours < 3 cm (n = 386; median age 9 years, IQR 7.5–11 years, range 1.5–17 years, *p* < 0.001). Malignant tumors were significantly larger (n = 686; median size 3.0 cm, IQR 1.5–5.0 cm, range 0.1–20.0 cm) than benign tumors (n = 137; median size 2.0 cm, IQR 1.0–3.0 cm, range 0.2–18 cm) and non-neoplastic lesions (n = 48; median size 2.0 cm, IQR 0.5–4.0 cm, range 0.3–7.5 cm; *p* < 0.001). There was no significant relationship between tumour size (< 3 cm vs. ≥ 3 cm) breed (*p* = 0.429), spayed status at mastectomy (intact vs. spayed) (*p* = 0.128) or timing of OH (*p* = 0.087). However, considering tumour size as a numerical variable (in cm), smaller tumours (median size 2.0 cm, IQR 1.5–4.3 cm, range 0.3–20 cm) were more often observed in intact compared with spayed bitches (median size 3.0 cm, IQR 1.5–4 cm, range 0.3–20 cm, *p* = 0.004).

#### Number and location of mammary tumours

Most dogs (73.4%, 1062/1447) had a single tumour, 26.6% of dogs (n = 385) had two or more tumours at the day of diagnosis (Table 4). CMTs described as multiple growths/diffuse pattern without distinct outlines, which could be difficult to count, were reported in 10.1% of dogs (n = 146), while separate, localized tumours (count from 1 to 8) occurred in 89.9% (n = 1301) of dogs. The association between spay status and the number of tumours, i.e. single vs. multiple (two or more tumours), was significant (*p* = 0.040). A significantly lower percentage of never spayed dogs had multiple CMTs (16.1%, 31/193) compared with single CMTs (27.3%, 71/260, *p* = 0.040). Considering dogs with multiple tumours, 80.3% (155/193) were hormonally active (intact and OH during mastectomy, OH < 1 year before), whereas 19.7% (38/193) were hormonally inactive. Multiple tumours were significantly smaller (median 2.5 cm, IQR 1–4 cm, range 0.3–20 cm) than single tumours (median 3.0 cm, IQR 2–5 cm, range 0.2–20 cm, *p* = 0.005). The number of CMTs was not significantly related to the age of the dogs (*p* = 0.236), tumour behaviour (*p* = 0.734), grade of carcinomas (*p* = 0.470), the presence of tumour necrosis (*p* = 0.305), or tumour ulceration (*p* = 0.585). There was no significant difference between single and multiple tumours with respect to the local recurrence (*p* = 0.810), lung metastases (*p* = 0.729), and CMT-SS (*p* = 0317). Slightly more CMTs were located on the left (51.7%, 422/816) than on the right side (40.9%, 334/816), followed by both-side location (7.4%, 60/816). The 4^th^–5^th^ glands (56.9%, 496/872) were most commonly affected followed by the 1^st^–2^nd^ glands (12.0%, 105/872).

#### Accuracy of lymphadenopathy at clinical examination in predicting metastases to the RLN

We analysed the accuracy of enlarged, palpable RLN in predicting metastases to these RLN (data were available only for 25 dogs). In 68% of cases (17/25), the histopathology of RLN was consistent with RLN enlargement. RLN metastases were detected by histopathology in 61.5% (8/13) of enlarged RLN and in 25.0% (3/12) of non-enlarged lymph nodes (Supplementary Table 6). In the diagnosis of RLN metastases, lymphadenopathy had a Se of 72.7% (95% CI 43.4%–90.3%) and a Sp of 64.3% (95% CI 38.8%–83.7%).

### Univariable and multivariable risk factor analysis of local recurrence, lung metastases, RLN metastases, CMT-specific survival, and CMT-related death.

#### Local recurrence

Eighty-six dogs with malignant CMTs and complete records were included in the full analysis (univariable and multivariable) for local recurrence of CMTs. Local recurrence developed in 11/86 dogs (12.8%; 95% CI 7.3%–21.5%). In univariable analysis, local recurrence of CMTs was significantly more common in dogs which had neoplastic emboli (OR 6.98, 95% CI 1.81%–26.9%, *p* = 0.006), tumour ulceration (OR 6.57, 95% CI 1.49%–29.0%, *p* = 0.021), simple carcinoma or complex carcinoma compared with other malignant subtypes (OR 7.05, 95% CI 0.86%–57.9%, *p* = 0.048) and metastasis to regional lymph node (OR 18.3, 95% CI 1.71%–196%, *p* = 0.016).

In multivariable analysis, the odds of local recurrence were significantly greater in dogs with neoplastic emboli (OR_adj_ 7.48, 95% CI 1.59%–35.2%, *p* = 0.011), tumour ulceration (OR_adj_ 6.23, 95% CI 1.00%–38.9%, *p* = 0.050), and simple carcinoma or complex carcinoma (OR_adj_ 10.9, 95% CI 1.09%–108%, *p* = 0.042). Metastasis to regional lymph node was no longer included in the multivariable model. (Table 5).

**Table 5:** Prognostic factors associated with local recurrence after mastectomy in dogs with malignant CMT (n = 86) tested by univariate and multivariate analysis.

Univariable analysis	Category	No. of dogs with lung metastases/no. of dogs in the category (%)	OR (95% CI)	*p* value
Neoplastic emboli	Yes	6/17 (35.3)	6.98 (1.81–26.9)	0.006*
	No	5/69 (7.3)		
Tumour ulceration	Yes	4/10 (40.0)	6.57 (1.49–29.0)	0.021*
	No	7/76 (.2)		
Histological subtype	Simple carcinoma & Complex carcinoma	10/54 (18.5)	7.05 (0.86–57.9)	0.048*
	Other malignant subtypes	1/32 (3.1)		
Metastases to RLN	Yes	3/12 (25.0)	18.3 (1.71–196)	0.016*
	No	1/56 (1.8)		

Multivariable analysis	Regression coefficient (SE)	Wald statistics	ORadj (95% CI)	*p* value
Intercept	− 4.43 (1.16)			
Simple carcinoma or complex carcinoma	2.38 (1.17)	4.13	10.9 (1.09–108)	0.042*
Neoplastic emboli	2.01 (0.79)	6.47	7.48 (1.59–35.2)	0.011*
Tumour ulceration	1.83 (0.93)	3.83	6.23 (1.00–38.9)	0.050^a^*

Hosmer&Lemeshow χ^2^ χ^2^ = 4.84, *p* = 0.184; Nagelkerke’s pseudo-R^2^ coefficient = 0.35,

^a^At the margin of significance,

CMT: canine mammary tumour; RLN: regional lymph nodes; OR: crude odds ratio; OR_adj_: adjusted odds ratio; CI confidence interval,

*Significant at *α* = *0.05*.

#### Lung metastases

Data on the prevalence of metastases during the follow-up period was available from 71 dogs with malignant CMTs. Lung metastases were observed in 18/71 dogs (25.4%) (95% CI 16.7%–36.6%). In univariable analysis, lung metastases were significantly more common in dogs older than 8 years (OR 4.47, 95% CI 0.93%–21.6%, *p* = 0.034) with tumour size ≥ 3 cm (OR 3.96, 95% CI 1.23%–12.8%, *p* = 0.016), carcinoma grade (OR 8.74, 95% CI 1.08%–71.1%, *p* = 0.026, especially grade II/III (OR 8.74, 95% CI 1.08%–71.1%, *p* = 0.026), and in dogs in which local recurrence (OR 19.1, 95% CI 3.40%–106%, *p* < 0.001) and metastasis to regional lymph node (OR 12.0, 95% CI 2.07%–69.7%, *p* = 0.009) were detected. In multivariable analysis, only the size of tumour was significant; dogs with tumours ≥ 3 cm exhibited fourfold increased odds of lung metastases compared with dogs with smaller tumours (< 3 cm) (OR_adj_ 3.96, 95% CI 1.23%–12.8%, *p* = 0.021), (Table 6).

**Table 6:** Prognostic factors associated with lung metastases after mastectomy in dogs with malignant CMT (n = 71) tested by univariable and multivariable analyses.

Univariable analysis	Category	No. of dogs with lung metastases/no. of dogs in the category (%)	OR (95% CI)	*p* value
Age > 8 years	Yes	16/50 (32.0)	4.47 (0.93–21.6)	0.034*
	No	2/21 (9.5)		
Tumour size	≥ 3 cm	13/34 (38.2)	3.96 (1.23–12.8)	0.016*
	< 3 cm	5/37 (13.5)		
Grade of carcinoma	I	1/19 (5.3)	–	0.026*
	II	9/31 (29.0)		
	III	8/21 (38.1)		
Grade II or III	Yes	17/52 (32.7)	8.74 (1.08–71.1)	0.028*
	No	1/19 (5.3)		
Local recurrence	Yes	7/9 (77.8)	19.1 (3.40–106)	< 0.001*
	No	9/58 (15.5)		
Metastases to RLN	Yes	4/7 (57.1)	12.0 (2.07–69.7)	0.009*
	No	5/50 (10.0)		

Multivariable analysis	Regression coefficient (SE)	Wald statistics	OR_adj_ (95% CI)	*p* value
Intercept	− 4.43 (1.16)			
Tumour size ≥ 3 cm	1.38 (0.60)	5.33	3.96 (1.23–12.8)	0.021*

CMT: canine mammary tumour; RLN: regional lymph nodes; OR: crude odds ratios; ORadj: adjusted odds ratios; CI confidence interval.

*Significant at α = 0.05.

### CMT-specific survival

Ninety dogs with malignant CMT and with complete records were included in the full analysis of CMT-SS with a median follow-up time of 20.0 months (IQR 14.2–26.0 months, range 1–53.6 months). Seventy (77.8%) patients were alive or died due to CMT-unrelated cause. Death in the perisurgical period was noted in 5 dogs (1 day in 4 dogs or 2 days in 1 dog after mastectomy), whereas 65 dogs were alive until being lost to follow up. A median follow-up time in dogs that survived (n = 65) was 24.3 months (IQR 18.3–28.4 months, range 0.5–53.6 months). In univariable analysis, the hazard of death was significantly higher in dogs spayed before mastectomy than in intact dogs at presentation for mastectomy (i.e. both spayed at mastectomy or never spayed, HR 2.59, 95% CI 1.07%–6.25%, *p* = 0.035), in dogs with tumour size ≥ 3 cm (HR 3.55, 95% CI 1.29%–9.82%, *p* = 0.015), multiple/diffuse tumours compared with separate/localized tumours (HR 3.90, 95% CI 1.40%–10.8%, *p* = 0.009), malignant CMT of increasing carcinoma grade (I vs. II vs. III, *p* = 0.007), especially in dogs with CMT grade III (HR 3.83, 95% CI 1.56%–9.39%, *p* = 0.003), tumour ulceration (HR 3.42, 95% CI 1.22%–9.59%, *p* = 0.019), and neoplastic emboli (HR 5.84, 95% CI 2.34%–14.6%, *p* < 0.001). Dogs with metastases to regional lymph nodes had shorter CMT-SS (median 7 months, IQR 2–18, range undefined, HR 22.2, 95% CI 7.00%–70.2%, *p* < 0.001), and a high risk of CMT-related death (OR 12.0, 95% CI 3.10%–46.5%, *p* < 0.001). In multivariable analysis, the hazard of death was significantly higher for dogs presented with multiple/diffuse tumours (HR_adj_ 4.82, 95% CI 1.62%–14.3%, *p* = 0.005), neoplastic emboli (HR_adj_ 5.23, 95% CI 2.06%–13.3%, *p* = 0.001), and tumour ulceration (HR_adj_ 3.97, 95% CI 1.32%–11.9%, *p* = 0.014), (Table 7).

**Table 7:** Prognostic factors associated with CMT-specific survival in dogs with malignant CMT (n = 90) tested by univariable and multivariable analysis.

Univariable analysis	Category	Median (IQR) SS [months]	HR (95% CI)	*p* value
Spay status at mastectomy	Spayed before mastectomy (n = 17)	Undefined (19 – undefined)	2.59 (1.07–6.25)	0.035*
	Intact (both spayed at mastectomy or left intact) (ref.) (n = 73)	Undefined (37 – undefined)		
Multiple tumours	Multiple/diffuse (n = 11)	22 (5 – undefined)	3.90 (1.40–10.8)	0.009*
	Localized tumours (ref.) (n = 79)	Undefined		
Tumour size	≥ 3 cm (n = 44)	37 (18 – undefined)	3.55 (1.29–9.82)	0.015*
	< 3 cm (ref.) (n = 46)	Undefined		
Neoplastic emboli	Yes (n = 17)	19 (2 – undefined)	5.84 (2.34–14.6)	< 0.001*
	No (ref.) (n = 73)	Undefined		
Tumour ulceration	Yes (n = 11)	19 (3 – 37)	3.42 (1.22–9.59)	0.019*
	No (ref.) (n = 79)	Undefined		
Grade of carcinoma	I (n = 20)	Undefined	–	0.007*
	II (n = 38)	Undefined		
	III (n = 32)	37 (9 – undefined)		
Grade III	Yes (n = 32)	37 (9 – undefined)	3.83 (1.56–9.39)	0.003*
	No (ref.) (n = 58)	Undefined		
Metastases to RLN	Yes (n = 10)	13 (2 – 18)	22.2 (7.00–70.2)	< 0.001*
	No (n = 41)	Undefined		

Multivariable analysis	Regression coefficient (SE)	χ^2^ statistics	HR_adj_ (95% CI)	*p* value
Multiple/diffuse tumours	1.57 (0.55)	8.01	4.82 (1.62–14.3)	0.005*
Neoplastic emboli	1.66 (0.48)	12.1	5.23 (2.06–13.3)	0.001*
Tumour ulceration	1.38 (0.56)	6.07	3.97 (1.32–11.9)	0.014*

RLN: regional lymph nodes; CMT: canine mammary tumour,

RLN: regional lymph nodes; HR: crude hazard ratios; HR_adj_: adjusted hazard ratios; CI confidence interval; **SS**: specific survival.

*Significant at *α* = *0.05.*

### CMT-related death

During the follow-up period, 20 dogs (22.2%, 20/90) with malignant CMT died due to CMT-related causes. Of these 20 dogs, 16 dogs developed lung metastases, 9 dogs – local recurrence, 4 dogs – RLN metastases, and 8 dogs – neoplastic emboli. The median CMT-SS was 10.8 months (IQR 4.0–17.2, range 0.5–36.5 months). In univariable analysis, CMT-related death was observed significantly more often in dogs spayed before mastectomy (HR 2.89, 95% CI 1.03%–8.07%, *p* = 0.043) compared with intact dogs (i.e. spayed at mastectomy or never spayed), in dogs with tumours ≥ 3 cm (HR 4.24, CI 95%: 1.39%–13.0%, *p* = 0.007), with neoplastic emboli (HR 4.52, 95% CI 1.45%–14.1%, *p* = 0.019), with malignant CMT of increasing grade (I vs. II vs. III; *p* = 0.013) – especially in dogs with grade III (HR 3.75, 95% CI 1.33%–10.5%, *p* = 0.011). CMT-related death was observed significantly more often in dogs with local recurrence (*p* < 0.001) and lung metastases (OR 400, 95% CI 34.0%–4708%, *p* < 0.001). In multivariable analysis, dog death caused by CMT was significantly associated with tumour size ≥ 3 cm (OR_adj_ 4.58, 95% CI 1.4%–14.9%, *p* = 0.011) and the presence of neoplastic emboli (OR_adj_ 4.96, 95% CI 1.46%–16.8%, *p* = 0.010) (Table 8).

**Table 8:** Prognostic factors for associated with CMT-related death tested by univariable and multivariable analysis. Twenty dogs out of 90 died due to malignant CMT-related cause.

Univariable analysis	Category	No. of dogs with CMT-related death/no. of dogs in the category (%)	OR (95% CI)	*p* value
Spay status at mastectomy	Spayed before mastectomy	10/28 (35.7)	2.89 (1.03–8.07)	0.043*
	Intact (both spayed at mastectomy or left intact)	10/62 (16.1)		
Tumour size	> 3 cm	15/44 (34.1)	4.24 (1.39–13.0)	0.007*
	≤ 3 cm	5/46 (10.9)		
Neoplastic emboli	Yes	8/17 (47.1)	4.52 (1.45–14.1)	0.019*
	No	12/73 (16.4)		
Grade	I	1/20 (5.0)	–	0.013*
	II	7/38 (18.4)		
	III	12/32 (37.5)		
Grade II or III	Yes	1/20 (5.0)	7.07 (0.89–56.6)	0.037*
	No	19/70 (27.1)		
Grade III	Yes	8/58 (13.8)	3.75 (1.33–10.5)	0.011*
	No	12/32 (37.5)		
Local recurrence	Yes	9/9 (100)	–	< 0.001*
	No	9/75 (12.0)		
Lung metastases	Yes	16/17 (94.1)	400 (34.0–4708)	< 0.001*
	No	2/52 (3.9)		
Metastases to RLN	Yes	8/14 (57.1)	12.0 (3.10–46.5)	< 0.001*
	No	6/60 (10.0)		

Multivariable analysis	Regression coefficient (SE)	Wald statistics	ORadj (95% CI)	*p* value
Intercept	− 2.55 (0.55)			
Tumour size ≥ 3 cm	1.52 (0.60)	6.40	4.58 (1.41–14.9)	0.011*
Neoplastic emboli	1.60 (0.62)	6.61	4.96 (1.46–16.8)	0.010*

Hosmer&Lemeshow χ^2^ test: χ^2^ = 0.31, *p* = 0.577; Nagelkerke’s pseudo-R^2^ coefficient = 0.22.

CMT: canine mammary tumour; RLN: regional lymph nodes; OR: crude odds ratio; OR_adj_: adjusted odds ratio; CI confidence interval.

*Significant at *α* = *0.05*.

## Discussion

### Age and breed

Out of 1447 female dogs, 83.3% were affected by malignant spontaneous CMTs and less frequently with benign (11.7%) or non-neoplastic lesions (5%)^6,7,45^. The majority of bitches with malignant CMTs revealed only this tumour behaviour and one histological subtype^19,46^. Moreover, they were frequently affected by simple carcinoma, grade II carcinoma^46,47^, and had a median age of 10 years at the day of tumour diagnosis, confirming previous reports^3,6,21,48,49^. The youngest dog was 1 year old, and the oldest was 17 years old. Some authors reported ages ranging from 1–2 to 20 years^23,50^ or from 1–3 to 15 years^51^. Similarly to other authors, we noted only one case in a one-year-old Dachshund diagnosed with a CMT^17^. Of note, the representation of the youngest dogs aged ≤ 5 years in our study was 5.8%. The prevalence of CMTs before 5 years of age is considered as rare, regardless of tumour behaviour^3,37^, and has previously been reported in only 1.52% of bitches under 4.8 years of age^9,52^. In the study from Sweden, 69 dogs with a CMT out of over 80,000 dogs were less than 3 years old^9^.

In the present study, dogs with a malignant and larger (≥ 3 cm) CMT were older (median age 10 years) than dogs with smaller tumours (< 3 cm, median age 9 years) and benign (median age 9 years) or non-neoplastic lesions (median age 8 years), which is in agreement with other authors who showed that malignant CMTs were significantly more frequent in older dogs with a mean age of 9.5 or 10.2 years compared with benign tumours in dogs with a mean age of 8.5 or 9.4 years, respectively^7,12,51,53,54^. This was in contrast to a previous study pointing that there was no significant difference between the age of dogs affected by benign tumours and malignant tumours^3,55^. Interestingly, young dogs aged ≤ 5 years were significantly more likely to develop a non-neoplastic lesion and/or a benign tumour compared with dogs over 5 years old^10,56^. Hence, we confirmed that the older dogs are more at risk of having a malignant CMT. Nevertheless, we failed to establish the age threshold which would be clinically useful for distinguishing between dogs with non-neoplastic/benign and malignant CMTs, emphasizing that the diagnosis of a malignant or non-neoplastic/benign lesion was independent from the age of the dogs^3^. In addition, although old age increases the risk of death from many diseases, it is questionable if age is a causative risk factor, because ageing is not a disease^57^.

In this study, smaller median tumour size was noted in intact dogs compared with spayed dogs, which was in agreement with a previous study^58^. In addition, benign tumours and non-neoplastic lesions were smaller compared with malignant tumours^58,59^. More recently, other authors noticed that the risk of having a malignant tumour increased approximately 1.5-fold with each 1.0 cm of increase in tumour size, while the risk increased approximately 11.8-fold when the tumour was larger than 5.0 cm compared with smaller tumours (< 3 cm)^60^. Although these data may suggest the previous theory of progression from benign to malignant with increasing tumour size, such an association has never been proven^12,59^.

Similar to our results, pedigree dogs in other studies were most frequently affected (72.8%), and they accounted for 59% to 80% of the study population^3,7,45,47,59^. However, some authors reported a higher proportion of mixed-breed dogs with CMTs^6,25,55,61^. In our study, regardless of tumour behaviour, Standard Dachshund and Yorkshire Terrier were most commonly affected among small-breed dogs, German Shepherd Dog and Boxer among large-breed dogs, and English Cocker Spaniel among medium-breeds^1,3,7,21,37,54,62^. Dogs of twelve overrepresented breeds and FCI group 4 (Dachshunds) were at high-risk for developing CMTs^50^ which may suggest a breed predilection to CMT. As in other studies, German Shepherd Dog was the second most frequent pedigree dog in the present study^3,9,53,55,63^. However, Beagle, Chihuahua, and Shih Tzu were poorly represented in contrast to some studies^17,49^. Additionally, FCI group 5 and group 9 demonstrated a decreased predisposition to CMTs. As far as we know, this is the first study to identify an association between FCI groups and risk of CMTs. Moreover, we demonstrated that some breeds had a high risk of a particular tumour behaviour, e.g. German Shepherd Dog and Standard Schnauzer were more likely to develop malignant tumours, while Chihuahuas, Jack Russell Terrier, and Labrador Retriever seemed to have a decreased predisposition to malignant CMTs^7,50,54,62^. Our results may reflect a great popularity of some breeds and regional variability. Therefore, the significant differences found may not reflect the genetic predisposition to CMT, and assessing breed predisposition in a local canine population can be misleading. On the other hand, the similarity of data from different countries suggests that the overrepresentation of some breeds may not necessarily be ‘just a coincidence’. Nevertheless, further research is still required^53,64^.

The strong association between large-breed dogs, young age of onset of CMT, and large size of CMT, regardless of tumour behaviour or presence of malignant CMTs, has been noticed.

These observations corroborate previous studies^3,6,65^; however, some authors did not find an association between benign and malignant CMTs or features attributed to malignancy (subtype and grade) and the size of a pedigree dog^3,40,47^. Our results may support the evidence that genetic diversity (different height/size category) influences the lifespan of pedigree dogs. Large-breed dogs have a shorter lifespan and an increased rate of aging, and hence may have more health problems, including malignant CMT, at a younger age compared with small-breed dogs^6,57^. In addition, when faced with healthcare costs in a shorter timeframe, owners may delay or discontinue treatment.

Although the high prevalence of malignant CMTs may actually reflect old age of the dog, age was not confirmed to be independent prognosticator. Importantly, some studies omitted age as a prognostic factor because old age itself has poorer prognosis associated with non-tumour factors such as co-morbidities^20^. The value of the height of a pedigree dog and overrepresentation of certain breeds to CMTs as prognostic factors was not confirmed in uni- and multivariable analysis.

### Hormonal status

Routine OH is often performed because of its protective value against reproductive tract disorders and CMTs. Depending on the age of the dog at the time of OH, potentially fatal CMTs may be preventable^14,17,18^. However, some studies did not confirm such a beneficial effect^15,66,67^. Discussions about the optimal age to spay and its effects have been going on for decades^68,69^. In the US and in the UK, early surgical neutering of dogs, e.g. before the age of 6 months for small-breed dogs and 12–18 months for large-breed dogs, became standard practice. In western European countries, the optimal time may be between the 1^st^ and 2^nd^ oestrus, when some protection against CMTs can be achieved, and some potential side-effects can be minimized^70,71^. On the contrary, there are hypotheses that OH performed in adult dogs may have a protective effect too, on CMT in general^18^ and even on benign CMT and non-neoplastic lesions^56,72^. Based on our survey, early spaying was less common in Poland. In line with previous studies, the majority of affected dogs were hormonally active at the day of CMT diagnosis^22,25,45^. This observation suggests that prolonged exposure of the mammary gland to sex-steroid hormones increases the prevalence of CMTs, confirming the protective effect of OH^17,18^. Unfortunately, we were unable to demonstrate any potential association between the age of the dog at OH and the risk of CMTs in general, because the exact data on the oestrus after which the bitch had OH in her youth was often not recorded, and because of the lack of simultaneous evaluation of the reference population^17,73^. In our survey, inactive dogs (spayed ≥ 1 year before mastectomy) were older than active ones (never spayed or spayed during mastectomy), which was in accordance with studies that reported a higher mean age of spayed dogs (10 years) compared with intact dogs (9 years)^7^. According to our results, hormonal status had no effect on the prevalence of malignant CMTs. Malignant CMTs often occurred equally in dogs regardless of OH and mastectomy time, most probably because the majority of bitches were spayed in late adulthood (median age of 10 years). Dogs spayed after the age of 2^1^/_2_ years are not protected against malignant CMTs, but only against benign CMTs. The risk of developing malignant CMT was the same as for an intact dogs^14^.

In our study, the increased time interval between OH and mastectomy was associated with the highest histological grade, which was often determined in bitches without sex hormone influence. Consequently, OH before mastectomy significantly reduced CMT-SS in dogs affected with malignant CMT and was more strongly associated with CMT-related deaths compared with hormonally active in univariable analysis. However, we could not confirm the independent prognostic value of OH conducted before mastectomy because of a small number of cases with complete information. Our observation was reinforced by previous studies which showed that spayed dogs were more often affected by highly malignant carcinomas compared with intact dogs, and that they had shorter disease-free survival after OH^18,40^. This could prove that malignant CMTs have a lower ER content than benign tumours, and will even have a decreasing ER expression as they progress towards more aggressive types with invasive and metastatic potential^13^. In contrast to our study, these reports did not analyse the timing of OH in relation to the mastectomy. Interestingly, other authors demonstrated that intact dogs or those spayed more than 2 years before mastectomy have shorter survival (median ~ 9 and 10 months, respectively) compared with dogs spayed less than 2 years before mastectomy (median ~ 24 months)^63^. According to the authors’ theory, a long interval between OH and mastectomy might promote ER-negative subtypes, which may correlate with poor prognosis^25,63^. Considering the dual role of oestrogen, its pro- and anti-cancer effects, as well as the spaying practices, further extensive research is needed^69,74^.

### Number and location of mammary tumours

Dogs affected with multiple mammary tumours are more common and accounted for 60.7%–82% in several studies^12,75,76^. However, in our study, the majority of dogs had one (73.4%) followed by two or more mammary tumours (26.6%), which was in line with recent results (61%–77% single vs. 23%–39% two or more)^40,55,77,78^. Some studies have shown a nearly equal incidence (45.6% single vs. 54.4% multiple)^59^. Multiple tumours were more common in hormonally active dogs, suggesting the effect of hormonal exposure on tumour multiplicity, potentially decreased by OH^54,58,59,72,77^, but one other report found no association^78^. On the other hand, the percentage of multiple tumours was significantly lower compared with single CMTs among intact bitches. This may suggest other factors, besides steroid hormones, influencing tumour multiplicity. There are still open questions as to whether CMTs develop separately as independent events or as a result of biological interactions between tumours (e.g. hormonal, genetic, autocrine, local spread from primary malignancy by lymphatic vessels)^12,77^. We found no association between the quantity of masses and the age of the dog. Although some studies have stated that multiple tumours were more frequent in old dogs, the mean age difference between dogs with multiple and single tumours was not large (10.1 years vs. 9.3 years, respectively)^54,58^.

The TNM staging system seems to be problematic in veterinary practice with regard to the selection of the conclusive tumour size (often attributed to the largest one) in multiple synchronous tumours. Multiple tumours are significantly smaller than a single mass^58,59^. This would fit the hypothesis that patients with multiple smaller tumours may be presented to the veterinary clinics earlier than with a single tumour.

To our knowledge, the present study evaluated for the first time the influence of not only the number of malignant tumours, but also of the presence of multiple/diffuse malignant tumours on survival outcomes. Diffuse involvement of multiple glands may appear as diffuse swelling with often unclear tumour boundaries^79^. On the other hand, we could not exclude that these tumours were uncountable due to other causes e.g. increased mammary adipose tissue in obese bitches. We demonstrated that dogs with multiple/diffuse malignant tumours have a higher risk of death compared with patients with a separated, easily localized tumour. This can be partially attributed to an infiltrative growth pattern or inflammatory mammary carcinoma without a defined separate palpable mass. We confirmed that the number of tumours had no effect on tumour behaviour, grade, tumour necrosis, tumour ulceration, and prognosis as each tumour may reveal different behaviour and grade^20,58,60,78^.

The majority of CMTs developed in the 4^th^ and 5^th^ glands, which is probably related to increased amount of glandular tissue and secretory activity of these mammae in dogs^23,25,47,51,55,80^. The left glands were more often affected, however, this observation should be considered incidental. We showed that the location of malignant CMTs did not affect prognosis^58,60,81,82^; however, in a recent study, dogs with the affected 1^st^ gland had a higher recurrence rate^58^.

### Accuracy of lymphadenopathy in predicting metastases to the RLN

The RLN status in dogs with CMTs has a prognostic value by itself and as a part of the TNM staging system^73,83^. When reviewing our database, we noted that RLN was quite frequently recorded as enlarged on preoperative clinical examination. Hence, we decided to determine the accuracy of lymphadenopathy in predicting RLN metastases in CMT patients. The diagnostic accuracy of physical examination for enlarged RLN was 68%, which offered no reliable value and was prone to producing certain false results^30,31,33^. Our findings were in line with previous data, even if a clinical examination was performed by a specialist surgeon^32,33^. The reasons for false positives were mainly related to reactive lymphoid hyperplasia, and those for false negatives were the presence of metastatic lesions in clinically non-palpable, non-enlarged RLNs, so RLN histopathology should be mandatory. Moreover, non-enlarged RLN are indistinguishable from subcutaneous adipose tissue, especially in obese bitches in which excess adipose tissue may mistakenly suggest swelling of this area^84^. Generally, RLN palpation can be challenging, difficult, requiring time and experience of the clinician. It is noteworthy that the nodal staging in CMT patients is not always defined on the sentinel lymph node in contrast to HBC patients, because the choice of sentinel lymph nodes in dogs is still challenging. A CMT can change the lymphatic drainage pattern by the formation of new lymphatic vessels, even leading to involvement of a large number of lymph nodes^80,85^. In veterinary medicine, sentinel lymph node mapping is not routinely performed and not considered as the gold standard^83^.

Although preoperative non-invasive testing of lymph node metastases has increased significantly in recent years in both human and veterinary medicine, the efficacy of clinical and imaging techniques varies and is still debated, and even controversial. Moreover, because of the low sensitivity of RLN palpation, cytology, and/or diagnostic imaging, the TNM staging system in dogs needs to be improved^27,86^.

### Outcome and Survival analysis

In the present study, analysis of local recurrence, lung metastases, CMT-SS, and CMT-related death was restricted to a small number of dogs and only those with malignant tumours. Of 90 female dogs, 22% died of their malignant CMTs, with previous reports ranging from 20%–31%^40,87^ to 54%–63%^17,25^. The presence of neoplastic emboli, tumour ulceration, and simple or complex carcinoma was demonstrated as independent predictors of local recurrence^21,88^. Although old age, large tumour size, grade II or III, and local recurrence were predictive of lung metastases in univariable analysis, only tumour size was retained as an independent prognostic factor^21,60,82^. This confirms that large malignant CMTs often need a long period of time to acquire metastatic potential^53^. We support the evidence that large tumour size, increasing histological grade, particularly grade III, tumour ulceration, and neoplastic emboli are related to shorter CMT-SS and/or CMT-related deaths, which corresponds to the previous results of univariable or multivariable analyses^83,88^. Corroborating previous CMT studies, histologically confirmed RLN metastases at diagnosis were associated with all negative outcomes: local recurrence, distant metastases, shorter CMT-SS, and CMT-related death. The latter was due to disease progression, the presence of local recurrence, neoplastic emboli, and local and/or lung metastases^25,89,90^. Nguyen et al.^25^ confirmed that pathologic nodal staging (pN) was a prognostic factor for overall survival and cancer-specific survival in dogs with invasive mammary carcinomas. However, in line with another study, we could not assess the prognostic value of RLN metastases due to an unsampled lymph node for histopathology^88^. In other studies, RLN metastases and (lympho)vascular invasion were combined into one group to avoid assessing a small sample due to the scarcity of available data on RLN status, or were assessed as a grading parameter in the Nottingham Prognostic Index (NPI)^21,27,87^.

The size of malignant tumour was related to specific survival only in univariable analysis; however, this parameter retained its independent prognostic power regarding CMT-related death. Dogs with a large malignant CMT have a nearly 4.6-fold increased risk of death compared with dogs with tumours smaller than 3 cm. This is reinforced by the previous observations that outcomes are significantly influenced by CMT size, but most previous studies are focused on OS and/or DFS, and only a few on specific survival (SS) and cancer-related death^25,27,40^. However, some studies are not comparable in terms of the method used to determine tumour size^25,27^. In our study, tumour size referred to the clinical size of the entire tumour based on gross measurements or after tumour excision. According to Chocteau et al.^27^, the clinical size of the tumour may be inaccurate because it may under- or overestimate its real size by taking into account the thickness of subcutaneous adipose tissue, possibly the adjacent hyperplastic lesion or additional nodules. It is still debatable if new subcategories (cut-offs) of clinical tumour size should be reevaluated for CMTs^12,40,58,59,82^. Based on HBC reports, pathologic tumour size (pT), determined by microscopic measurement on H-E histological slides has been proposed despite some limitations. Nevertheless, due to the discrepancy between clinical tumour size and pT caused by observer-dependent and technical factors, it seems that the most accurate size should use information at the time of clinical and microscopic examination^25,27,91^.

Neoplastic emboli, described here, were a predictor of shorter CMT-SS and of death of dogs with malignant CMT^25,27,49,87^, whereas histological grade lost its prognostic value, which was similar to some multivariable studies^21,48,88,90^. However, Pastor et al.^92^ proved the prognostic value of peritumoural invasion (the presence of neoplastic cells infiltrating the normal tissue adjacent to the tumour) but not vascular invasion by neoplastic emboli.

Consistent with our results, skin ulceration over a malignant CMT has been proposed as a﻿ prognostic value of CMT-SS^27,93^. In HBC studies, skin involvement (referred to as ulceration, oedema, peau d'orange, and satellite skin nodules) is included in the TNM classification despite some discrepancies regarding its prognostic value^94^. In light of our findings, we believe that this feature should be considered for CMT evaluation and in the future revision of TNM staging system.

## Study limitations

The present study has some limitations as in most retrospective investigations. Firstly, the small amount of data on the timing of OH, reproductive clinical history, and complete TNM cases for the full risk factor analysis (univariable and multivariable). It could be due to the fact that cover letters for histopathology were more often filled out by a surgical specialist than by a primary care veterinarian. On the other hand, referral templates have changed over the years and most of them were not specifically designed to gather detailed information on reproductive health. Secondly, a relatively small sample size was referred to the RLN tested for histopathology, and finally, in most cases, the necessary complete 2-year follow-up information was lost or unavailable. Furthermore, our results were not compared with a control population without CMTs. Each dog was counted only once even if it appeared several times in our database over the years. Regardless of our effort to not repeat a case and overestimate the number of dogs, we could only rely on the comprehensiveness of submission letters. This was a single-institution study recruiting diagnosed dogs living in central Poland and it did not strictly reflect the prevalence of female dogs with CMTs across the country.

## Conclusions

This study confirms the previously published data on dogs with CMTs with respect to age, breed, spay status, tumour behaviour and size, as well as number and location of tumours. It provides the first evidence of CMT risk for FCI groups, a low diagnostic accuracy of RLN palpation in preoperative examination and gives clinically relevant information on the timing of ovariohysterectomy, independent predictors of local recurrence, local and/or lung metastases, CMT-specific survival, and CMT-related death. Despite the low completeness of the 2-year follow-up information in the study, it is the first survival analysis of female dogs after mastectomy in Poland on such a scale, which was possible thanks to the veterinarians’ and, therefore, dog owners’ greater awareness of the importance of long-term follow-up in veterinary research. Undoubtedly, a canine cancer registry in Poland would increase the availability of data.

### Supplementary Information

Supplementary Tables.

## Data Availability

The data generated and analysed in this study are included in this published article (and its Supplementary Information files). Other datasets are available from the corresponding author on a reasonable request.
