# Supplementary material for: Risk factor analysis and clinicopathological characteristics of female dogs with mammary tumours from a single-center retrospective study in Poland
Source: Sci Rep. 2024 Mar 6;14:5569. doi: 10.1038/s41598-024-56194-z (PMC10917774; doi:10.1038/s41598-024-56194-z)
Supplement: Supplementary file 1 — Supplementary Tables. [file 41598_2024_56194_MOESM1_ESM.docx]

**Risk factor analysis and clinicopathological characteristics of female dogs with mammary tumours from a single-center retrospective study in Poland**

Izabella Dolka^1,^*, Michał Czopowicz^2^, Diana Stopka^1^, Agata Wojtkowska^3^,

Ilona Kaszak^3^, Rafał Sapierzyński^1^

^1^Department of Pathology and Veterinary Diagnostics, Institute of Veterinary Medicine, Warsaw University of Life Sciences (SGGW), Nowoursynowska 159c, 02-776 Warsaw, Poland

^2^Division of Veterinary Epidemiology and Economics, Institute of Veterinary Medicine, Warsaw University of Life Sciences (SGGW), Nowoursynowska 159c, 02-776 Warsaw, Poland

^3^Department of Small Animal Diseases with Clinic, Institute of Veterinary Medicine, Warsaw University of Life Sciences (SGGW), Nowoursynowska 159c, 02-776 Warsaw, Poland

*Corresponding author: Izabella Dolka, izabella_dolka@sggw.edu.pl; Department of Pathology and Veterinary Diagnostics, Institute of Veterinary Medicine, Warsaw University of Life Sciences (SGGW), Nowoursynowska 159c, 02-776 Warsaw, Poland

**Supplementary Table 1.** The association between the age of the dog and tumour behaviour: non-neoplastic mammary lesions, benign CMT and malignant CMT.

|  | **Non-neoplastic (n=65)** | **Benign (n=167)** | **Non-neoplastic or benign together (n=232)** | **Malignant (n=1139)** | **NN vs. B vs. M**  **P-value** | **NN/B vs. M**  **P-value** |
| --- | --- | --- | --- | --- | --- | --- |
| Age  [median, IQR (range)] | 8, 7–10 (2.5–14.5) | 9, 7–10  (2–16) | 9, 7–10  (2–16) | 10, 8–12  (1–17) | NN vs. B p=0.999  NN vs. M 0.002*  B vs. M <0.001* | <0.001* |

CMT – canine mammary tumors

NN – non-neoplastic mammary lesion,
B – benign mammary tumour,
M – malignant mammary tumour
IQR – interquartile range,
* significant at α=0.05.

**Supplementary Table 2.** The comparison of the prevalence of tumour behaviour in dogs less than and equal to 5 years of age and more than 5 years of age. Results are presented as n(%) of dogs (with 95% CI ).

|  | **Age class  [n (% from age class; 95% CI)]** | |  |
| --- | --- | --- | --- |
| **Lesion** | **≤ 5 years (N=79)** | **> 5 years (N=1292)** | **p-value** |
| Non-neoplastic | 7 (8.9; 4.4–17.2) | 58 (4.5; 3.5–5.8) | 0.076 |
| Benign | 18 (22.8; 14.9–33.2) | 149 (11.5; 9.9–13.4) | 0.003* |
| Non-neoplastic or benign together | 25 (31.6; 22.4–42.5) | 207 (16.0; 14.1–18.1) | <0.001* |
| Malignant | 54 (68.4; 57.5–77.6) | 1085 (84.0; 81.9–85.9) | <0.001* |

CI 95% – 95% confidence interval,

* significant at α=0.05.

**Supplementary Table 3.** List of pedigree dogs with known breed (n=1006) with CMT as well as their numbers and frequencies.

|  | **Breed** | **N** | **%** |
| --- | --- | --- | --- |
| 1. | Mixed-breed | 375 | 27.2% |
| 2. | Pedigree | 1006 | 72.8% |
|  | **Pedigree dogs** | **N** | **%=n/1006** |
| 1. | Standard Dachshund | 194 | 19.3% |
| 2. | German Shepherd Dog | 142 | 14.1% |
| 3. | Yorkshire Terrier | 95 | 9.4% |
| 4. | Boxer | 50 | 5.0% |
| 5. | English Cocker Spaniel | 42 | 4.2% |
| 6. | Miniature Poodle | 34 | 3.4% |
| 7. | Doberman | 32 | 3.2% |
| 8. | Standard Schnauzer | 28 | 2.8% |
| 9. | Miniature Schnauzer | 26 | 2.6% |
| 10. | American Staffordshire Terrier | 22 | 2.2% |
| 11. | Golden Retriever | 20 | 2.0% |
| 12. | Rottweiler | 18 | 1.8% |
| 13. | Miniature Pinscher | 17 | 1.7% |
| 14. | Giant Schnauzer | 15 | 1.5% |
| 15. | Fox Terrier | 14 | 1.4% |
| 16. | Weimaraner | 12 | 1.2% |
| 17. | Bavarian Mountain Scent Hound | 12 | 1.2% |
| 18. | Beagle | 11 | 1.1% |
| 19. | Labrador Retriever | 11 | 1.1% |
| 20. | French Bulldog | 9 | 0.9% |
| 21. | Medium Poodle | 8 | 0.8% |
| 22. | Irish Red Setter | 7 | 0.7% |
| 23. | Collie (Rough) | 7 | 0.7% |
| 24. | Pekingese | 7 | 0.7% |
| 25. | Great Dane | 6 | 0.6% |
| 26. | Shih Tzu | 6 | 0.6% |
| 27. | Black Russian Terrier | 6 | 0.6% |
| 28. | West Highland White Terrier | 6 | 0.6% |
| 29. | Jack Russel Terrier | 5 | 0.5% |
| 30. | Italian Sighthound | 5 | 0.5% |
| 31. | Gordon Setter | 5 | 0.5% |
| 32. | Alaskan Malamute | 5 | 0.5% |
| 33. | Dalmatian | 5 | 0.5% |
| 34. | Staffordshire Bull Terrier (Stafford) | 5 | 0.5% |
| 35. | Polish Hunting Dog | 4 | 0.4% |
| 36. | Siberian Husky | 4 | 0.4% |
| 37. | Polish Lowland Sheepdog | 4 | 0.4% |
| 38. | Pembroke Welsh Corgi | 4 | 0.4% |
| 39. | Akita, Akita Inu | 4 | 0.4% |
| 40. | Chihuahua | 4 | 0.4% |
| 41. | American Pit Bull Terrier | 3 | 0.3% |
| 42. | Bull Terrier | 3 | 0.3% |
| 43. | Bedlington Terrier | 3 | 0.3% |
| 44. | Briard | 3 | 0.3% |
| 45. | Airedale Terrier | 3 | 0.3% |
| 46. | Welsh Terrier | 3 | 0.3% |
| 47. | German Wirehaired Pointer | 3 | 0.3% |
| 48. | Whippet | 3 | 0.3% |
| 49. | Rhodesian Ridgeback | 3 | 0.3% |
| 50. | Afghan Hound | 2 | 0.2% |
| 51. | Old English Sheepdog (Bobtail) | 2 | 0.2% |
| 52. | Bullmastiff | 2 | 0.2% |
| 53. | Bouvier des Flandres | 2 | 0.2% |
| 54. | Scottish Terrier | 2 | 0.2% |
| 55. | Irish Red Terrier | 2 | 0.2% |
| 56. | Caucasian Shepherd Dog | 2 | 0.2% |
| 57. | Bloodhound | 2 | 0.2% |
| 58. | American Akita | 2 | 0.2% |
| 59. | Central Asia Shepherd Dog | 2 | 0.2% |
| 60. | English Bulldog | 2 | 0.2% |
| 61. | St.Bernard | 2 | 0.2% |
| 62. | Polish Hound | 2 | 0.2% |
| 63. | Hovawart | 2 | 0.2% |
| 64. | Tatra Shepherd Dog | 2 | 0.2% |
| 65. | Cavalier King Charles Spaniel | 2 | 0.2% |
| 66. | Maltese | 2 | 0.2% |
| 67. | Dog de Bordeaux | 1 | 0.1% |
| 68. | Leonberger | 1 | 0.1% |
| 69. | Tosa, Tosa Inu | 1 | 0.1% |
| 70. | Cairn Terrier | 1 | 0.1% |
| 71. | Irish soft Coated Wheaten Terrier | 1 | 0.1% |
| 72. | German Giant Spitz | 1 | 0.1% |
| 73. | Tibetan Terrier | 1 | 0.1% |
| 74. | Russian-European Laika | 1 | 0.1% |
| 75. | Flat Coated Retriever | 1 | 0.1% |
| 76. | Basset Hound | 1 | 0.1% |
| 77. | American Cocker Spaniel | 1 | 0.1% |
| 78. | German Pinscher | 1 | 0.1% |
| 79. | German Hunting Terrier, Deutcher Jagdterier | 1 | 0.1% |
| 80. | Bichon Frisé | 1 | 0.1% |
| 81. | Newfoundland | 1 | 0.1% |
| 82. | Norwich Terrier | 1 | 0.1% |
| 83. | Lhasa Apso | 1 | 0.1% |
| 84. | Great Swiss Mountain Dog | 1 | 0.1% |
| 85. | Shar Pei | 1 | 0.1% |
| 86. | Continental Toy Spaniel (Papillon) | 1 | 0.1% |
| 87. | Australian Terrier | 1 | 0.1% |
| 88. | Bohemian Wire-Haired Pointing Griffon (Cesky Fousek) | 1 | 0.1% |
| 89. | English Springer Spaniel | 1 | 0.1% |
| 90. | Russian Spaniel | 1 | 0.1% |
| 91. | Beauceron | 1 | 0.1% |
| 92. | Pug | 1 | 0.1% |
| 93. | Presa Canario | 1 | 0.1% |
| 94. | English Setter (Laverack) | 1 | 0.1% |
| 95. | Border Collie | 1 | 0.1% |
| 96. | Tibetan Mastiff | 1 | 0.1% |
| 97. | Czechoslovakian Wolfdog | 1 | 0.1% |
| 98. | Irish Wolfhound | 1 | 0.1% |
| 99. | Italian Cane Corso | 1 | 0.1% |
| 100. | Borzoi – Russian Hunting Sighthound | 1 | 0.1% |

**Supplementary** **Table. 4**. Relationship between breed and the occurrence of CMTs in pedigree dogs (n=1006) based on the theoretical distribution of Polish pedigree dogs based on registers of the Polish Kennel Club from years 2009–2019.

| **Breed** | **CMT [n(%)]** | | **OR (95% CI)** | **P-value** |
| --- | --- | --- | --- | --- |
|  | **Female pedigree dogs affected with CMT (n=1006)** | **Polish theoretical distribution of 1000 female pedigree dogs** |  |  |
| Standard Dachshund^a^ | 194 (19.3) | 28 (2.8) | 8.29 (5.52–12.5) | <0.001* |
| German Shepherd Dog^a^ | 142 (14.1) | 86 (8.6) | 1.75 (1.32–2.32) | <0.001* |
| Yorkshire Terrier^a^ | 95 (9.4) | 50 (5.0) | 1.98 (1.39–2.82) | <0.001* |
| Boxer^a^ | 50 (5.0) | 8 (0.8) | 6.49 (3.06–13.8) | <0.001* |
| English Cocker Spaniel^a^ | 42 (4.2) | 7 (0.7) | 6.18 (2.76–13.8) | <0.001* |
| Miniature Poodle^a^ | 34 (3.4) | 4 (0.4) | 8.71 (3.08–24.6) | <0.001* |
| Doberman^a^ | 32 (3.2) | 6 (0.6) | 5.44 (2.27–13.1) | <0.001* |
| Standard Schnauzer^a^ | 28 (2.8) | 3 (0.3) | 9.51 (2.88–31.4) | <0.001* |
| Miniature Schnauzer | 26 (2.6) | 19 (1.9) | 1.37 (0.75–2.49) | 0.377 |
| American Staffordshire Terrier | 22 (2.2) | 13 (1.3) | 1.70 (0.85–3.39) | 0.178 |
| Golden Retriever | 20 (2.0) | 22 (2.2) | 0.90 (0.49–1.66) | 0.861 |
| Rottweiler | 18 (1.8) | 10 (1.0) | 1.80 (0.83–3.93) | 0.188 |
| Miniature Pinscher^a^ | 17 (1.7) | 4 (0.4) | 4.28 (1.44–12.8) | 0.009* |
| Giant Schnauzer^a^ | 15 (1.5) | 5 (0.5) | 3.01 (1.09–8.32) | 0.045* |
| Fox Terrier^a^ | 14 (1.4) | 4 (0.4) | 3.51 (1.15–10.7) | 0.034* |
| Weimaraner | 12 (1.2) | 6 (0.6) | 2.00 (0.75–5.35) | 0.242 |
| Bavarian Mountain  Scent Hound | 12 (1.2) | 8 (0.8) | 1.50 (0.61–3.68) | 0.509 |
| Beagle | 11 (1.1) | 12 (1.2) | 0.91 (0.40–2.07) | 0.989 |
| Labrador Retriever^b^ | 11 (1.1) | 40 (4.0) | 0.27 (0.14–0.52) | <0.001* |
| French Bulldog^b^ | 9 (0.9) | 29 (2.9) | 0.30 (0.14–0.64) | 0.002* |
| Medium Poodle^a^ | 9 (0.9) | 1 (0.1) | 9.02 (1.14–71.3) | 0.027* |
| Irish Red Setter | 8 (0.8) | 5 (0.5) | 1.60 (0.52–4.89) | 0.585 |
| Collie (Rough) | 7 (0.7) | 6 (0.6) | 1.16 (0.39–3.47) | 0.991 |
| Pekingese | 7 (0.7) | 1 (0.1) | 7.00 (0.86–57.0) | 0.078 |
| Great Dane | 7 (0.7) | 13 (1.3) | 0.53 (0.21–1.34) | 0.256 |
| Shih Tzu | 6 (0.6) | 8 (0.8) | 0.74 (0.26–2.15) | 0.780 |
| Black Russian Terrier | 6 (0.6) | 6 (0.6) | 0.99 (0.32–3.09) | 0.780 |
| West Highland White Terrier^b^ | 6 (0.6) | 16 (1.6) | 0.37 (0.14–0.95) | 0.031* |
| Jack Russel Terrier | 6 (0.6) | 15 (1.5) | 0.39 (0.15–1.02) | 0.077 |
| Italian Sighthound | 5 (0.5) | 4 (0.4) | 1.24 (0.33–4.65) | 0.993 |
| Gordon Setter | 5 (0.5) | 3 (0.3) | 1.66 (0.40–6.96) | 0.730 |
| Alaskan Malamute | 5 (0.5) | 6 (0.6) | 0.83 (0.25–2.72) | 0.992 |
| Dalmatian | 5 (0.5) | 1 (0.1) | 4.99 (0.58–42.8) | 0.223 |
| Staffordshire Bull Terrier | 5 (0.5) | 11 (1.1) | 0.45 (0.16–1.30) | 0.205 |
| Polish Hunting Dog^b^ | 4 (0.4) | 13 (1.3) | 0.30 (0.10–0.93) | 0.024* |
| Siberian Husky^b^ | 4 (0.4) | 13 (1.3) | 0.30 (0.10–0.93) | 0.024* |
| Polish Lowland Sheepdog | 4 (0.4) | 6 (0.6) | 0.66 (0.19–2.35) | 0.519 |
| Pembroke Welsh Corgi | 4 (0.4) | 3 (0.3) | 1.33 (0.30–5.94) | 0.710 |
| Akita, Akita Inu | 4 (0.4) | 8 (0.8) | 0.50 (0.15–1.65) | 0.238 |
| Chihuahua^b^ | 4 (0.4) | 37 (3.7) | 0.10 (0.04–0.29) | <0.001* |
| American Pit Bull Terrier^c^ | 3 (0.3) |  |  |  |
| Bull Terrier^b^ | 3 (0.3) | 12 (1.2) | 0.25 (0.07–0.88) | 0.015 |
| Bedlington Terrier | 3 (0.3) | 1 (0.1) | 2.99 (0.31–28.8) | 0.309 |
| Briard | 3 (0.3) | 3 (0.3) | 0.99 (0.20–4.94) | 0.994 |
| Airedale Terrier | 3 (0.3) | 4 (0.4) | 0.74 (0.17–3.34) | 0.699 |
| Welsh Terrier | 3 (0.3) | 4 (0.4) | 0.74 (0.17–3.34) | 0.699 |
| German Wirehaired Pointer | 3 (0.3) | 2 (0.2) | 1.49 (0.25–8.95) | 0.658 |
| Whippet | 3 (0.3) | 7 (0.7) | 0.42 (0.11–1.65) | 0.195 |
| Rhodesian Ridgeback | 3 (0.3) | 4 (0.4) | 0.74 (0.17–3.34) | 0.699 |
| Afghan Hound | 2 (0.2) | 1 (0.1) | 1.99 (0.18–22.0) | 0.563 |
| Old English Sheepdog (Bobtail) | 2 (0.2) | 1 (0.1) | 1.99 (0.18–22.0) | 0.563 |
| Bullmastiff | 2 (0.2) | 2 (0.2) | 0.99 (0.14–7.07) | 0.995 |
| Bouvier des Flandres | 2 (0.2) | 1 (0.1) | 1.99 (0.18–22.0) | 0.563 |
| Scottish Terrier | 2 (0.2) | 4 (0.4) | 0.50 (0.09–2.71) | 0.405 |
| Irish Red Terrier | 2 (0.2) | 1 (0.1) | 1.99 (0.18–22.0) | 0.563 |
| Caucasian Shepherd Dog | 2 (0.2) | 6 (0.6) | 0.33 (0.07–1.64) | 0.145 |
| Bloodhound | 2 (0.2) | 1 (0.1) | 1.99 (0.18–22.0) | 0.563 |
| American Akita | 2 (0.2) | 4 (0.4) | 0.50 (0.09–2.71) | 0.405 |
| Central Asia Shepherd Dog^b^ | 2 (0.2) | 10 (1.0) | 0.20 (0.04–0.90) | 0.015 |
| English Bulldog^b^ | 2 (0.2) | 18 (1.8) | 0.11 (0.03–0.47) | <0.001* |
| St.Bernard | 2 (0.2) | 7 (0.7) | 0.28 (0.06–1.36) | 0.084 |
| Polish Hound | 2 (0.2) | 6 (0.6) | 0.33 (0.07–1.64) | 0.145 |
| Hovawart | 2 (0.2) | 9 (0.9) | 0.22 (0.05–1.02) | 0.068 |
| Tatra Shepherd Dog | 2 (0.2) | 1 (0.1) | 1.99 (0.18–22.0) | 0.563 |
| Cavalier King Charles Spaniel^b^ | 2 (0.2) | 18 (1.8) | 0.11 (0.03–0.47) | <0.001* |
| Maltese^b^ | 2 (0.2) | 12 (1.2) | 0.16 (0.04–0.73) | 0.005 |
| Dog de Bordeaux | 1 (0.1) | 4 (0.4) | 0.25 (0.03–2.22) | 0.367 |
| Leonberger | 1 (0.1) | 5 (0.5) | 0.20 (0.02–1.70) | 0.217 |
| Tosa, Tosa Inu | 1 (0.1) | 3 (0.3) | 0.33 (0.03–3.18) | 0.613 |
| Cairn Terrier | 1 (0.1) | 3 (0.3) | 0.33 (0.03–3.18) | 0.613 |
| Irish Soft Coated Wheaten Terrier | 1 (0.1) | 1 (0.1) | 0.99 (0.06–15.9) | 0.997 |
| Tibetan Terrier | 1 (0.1) | 1 (0.1) | 0.99 (0.06–15.9) | 0.997 |
| Russian-European Laika | 1 (0.1) | 2 (0.2) | 0.50 (0.04–5.48) | 0.996 |
| Flat Coated Retriever | 1 (0.1) | 5 (0.5) | 0.20 (0.02–1.70) | 0.217 |
| Basset Hound^b^ | 1 (0.1) | 8 (0.8) | 0.12 (0.02–0.99) | 0.044* |
| American Cocker Spaniel | 1 (0.1) | 1 (0.1) | 0.99 (0.06–15.9) | 0.997 |
| German Pinscher | 1 (0.1) | 7 (0.7) | 0.14 (0.02–1.15) | 0.075 |
| German Hunting Terrier, (Deutcher Jagdterier) | 1 (0.1) | 7 (0.7) | 0.14 (0.02–1.15) | 0.075 |
| Bichon Frisé | 1 (0.1) | 3 (0.3) | 0.33 (0.03–3.18) | 0.303 |
| Newfoundland | 1 (0.1) | 7 (0.7) | 0.14 (0.02–1.15) | 0.075 |
| Norwich Terrier | 1 (0.1) | 1 (0.1) | 0.99 (0.06–15.9) | 0.997 |
| Lhasa Apso | 1 (0.1) | 4 (0.4) | 0.25 (0.03–2.22) | 0.367 |
| Great Swiss Mountain Dog | 1 (0.1) | 4 (0.4) | 0.25 (0.03–2.22) | 0.367 |
| Shar Pei | 1 (0.1) | 4 (0.4) | 0.25 (0.03–2.22) | 0.367 |
| Continental Toy Spaniel (Papillon) | 1 (0.1) | 6 (0.6) | 0.16 (0.02–1.37) | 0.128 |
| Australian Terrier | 1 (0.1) | 1 (0.1) | 0.99 (0.06–15.9) | 0.997 |
| Bohemian Wire-Haired Pointing Griffon (Cesky Fousek) | 1 (0.1) | 1 (0.1) | 0.99 (0.06–15.9) | 0.997 |
| English Springer Spaniel | 1 (0.1) | 3 (0.3) | 0.33 (0.03–3.18) | 0.303 |
| Russian Spaniel^c^ | 1 (0.1) |  |  |  |
| Beauceron | 1 (0.1) | 1 (0.1) | 0.99 (0.06–15.9) | 0.997 |
| Pug^b^ | 1 (0.1) | 12 (1.2) | 0.08 (0.01–0.63) | 0.001* |
| Presa Canario | 1 (0.1) | 1 (0.1) | 0.99 (0.06–15.9) | 0.997 |
| English Setter (Laverack) | 1 (0.1) | 2 (0.2) | 0.50 (0.04–5.48) | 0.556 |
| Border Collie^b^ | 1 (0.1) | 13 (1.3) | 0.08 (0.01–0.58) | <0.001* |
| Tibetan Mastiff | 1 (0.1) | 7 (0.7) | 0.14 (0.02–1.15) | 0.075 |
| Czechoslovakian Wolfdog | 1 (0.1) | 3 (0.3) | 0.33 (0.03–3.18) | 0.303 |
| Irish Wolfhound | 1 (0.1) | 2 (0.2) | 0.50 (0.04–5.48) | 0.556 |
| Italian Cane Corso^b^ | 1 (0.1) | 10 (1.0) | 0.10 (0.01–0.77) | 0.003* |
| Borzoi – Russian Hunting Sighthound | 1 (0.1) | 3 (0.3) | 0.33 (0.03–3.18) | 0.303 |

^a^ Breeds significantly overrepresented (suspected predisposition to CMT),

^b^ Breeds significantly under-represented,
^c^ Not recognized as a breed by FCI,
CMT – canine mammary tumour,
OR – crude odds ratios,
CI – confidence interval,
* significant at α=0.05.

**Supplementary Table 5.** The comparison of the age of the dog at time of OH between dogs with non-neoplastic mammary lesions, benign CMT and malignant CMT (335 dogs included).

| **Type of CMT (N)** | **The age of the dog at OH**  **[median, IQR (range)]** | **P-value** |
| --- | --- | --- |
| Benign or non-neoplastic (73) | 9, 7–10 (2–16) | 0.318 |
| Malignant (262) | 9, 7–11 (0.5–15.6) |  |
|  | |  |
| Non-neoplastic (22) | 10, 7– 11 (3–13) | 0.566 |
| Benign (51) | 9, 7– 10 (2–16) |  |
| Malignant (262) | 9, 7–11 (0.5–15.6) |  |

CMT – canine mammary tumour,
OH – ovariohysterectomy,
IQR – interquartile range,
* significant at α=0.05.

**Supplementary Table 6.** Accuracy of the regional lymphadenopathy (enlargement of the lymph node) with histopathology in the diagnosis of regional lymph node (RLN) metastases.

| **Palpation of RLN** | **Metastasis to the RLN  Histopathology** | | **Totals** |
| --- | --- | --- | --- |
|  | **Positive** | **Negative** |  |
| **Positive** | 8 | 5 | 13 |
| **Negative** | 3 | 9 | 12 |
| Totals | 11 | 14 | 25 |
